# Research progress on precision orchard yield estimation based on multi-source information perception sensors

**DOI:** 10.3389/fpls.2026.1759956

**Published:** 2026-04-13

**Authors:** Zifan Rong, Yu Ru, Xiao Zhang, Shuping Fang, Hongping Zhou, Xuesong Jiang

**Affiliations:** 1 College of Mechanical and Electronic Engineering, Nanjing Forestry University, Nanjing, China; 2 School of Artificial Intelligence, Zhoukou Normal University, Zhoukou, China

**Keywords:** crop monitoring, data heterogeneity, deep learning, precision agriculture, yield mapping

## Abstract

**Introduction:**

Accurate orchard yield data are essential for economic assessment and management optimization, but traditional manual estimation is labor-intensive and often inaccurate for modern precision orchard management.

**Methods:**

Following the core principles of the PRISMA guidelines, this systematic review summarizes recent domestic and international studies on orchard yield estimation and compares machine vision, remote sensing, and multi-source data fusion methods from both methodological and application perspectives.

**Results:**

Existing studies show that machine vision and remote sensing can effectively support automated orchard yield estimation, while multi-source heterogeneous data fusion generally improves robustness and estimation accuracy by integrating fruit detection information with canopy physiological and structural traits.

**Discussion:**

Despite rapid progress, major challenges remain, including fruit occlusion and missed detection, data heterogeneity, and limited automation and adaptability across orchard scenarios. Future work should strengthen advanced algorithm integration, promote multi-modal data fusion, and develop intelligent, automated yield-estimation platforms for diverse orchard environments.

## 1 Introduction

Yield estimation is a core component of orchard production management, providing crucial data support for decision-making related to fertilization, storage, and marketing (Farooqui et al., 2024). Traditional orchard yield estimation methods primarily rely on manual surveys and empirical assessments, which present significant limitations for large-scale yield estimation. These methods are not only labor-intensive and time-consuming but also fail to fully account for individual tree differences and the influence of environmental factors such as topography and light conditions on fruit development, resulting in considerable estimation errors.

With the rapid development of machine vision and remote sensing technologies, modern methods for orchard yield estimation have gradually emerged, showing promise in overcoming the shortcomings of traditional approaches. However, current research in this field is still at an early exploratory stage, lacking unified and systematic technical standards and frameworks. Therefore, establishing an automated, high-precision orchard yield estimation system to enhance data reliability and applicability is of great significance for promoting the modernization of the fruit industry. In the field of orchard yield estimation, early studies often constructed empirical regression models by integrating meteorological, topographical, and other environmental factors to predict regional orchard yields through statistical analysis (Paltineanu and Chitu, 2006; Ubalde et al., 2007). Nevertheless, since orchard yield is influenced by a variety of factors including climate, management practices, and varietal differences, estimation models based solely on ecological and environmental parameters generally lack robustness and generalization in real-world applications, especially when faced with extreme weather events or unexpected incidents.

Recent research trends have shifted towards precision yield estimation methods that incorporate physiological characteristics and biological data of fruit trees (Kwon et al., 2024). These studies employ a range of information perception sensors—such as machine vision systems, hyperspectral cameras, and LiDAR—to collect data on tree morphology, physiological status, and fruit quantity, followed by algorithmic analysis and modeling to achieve refined yield prediction at either the individual tree or regional scale (Kang and Chen, 2020; Llorens et al., 2020). Compared with traditional approaches, sensor-based estimation methods can provide more accurate and targeted predictions.

**Figure 1:**
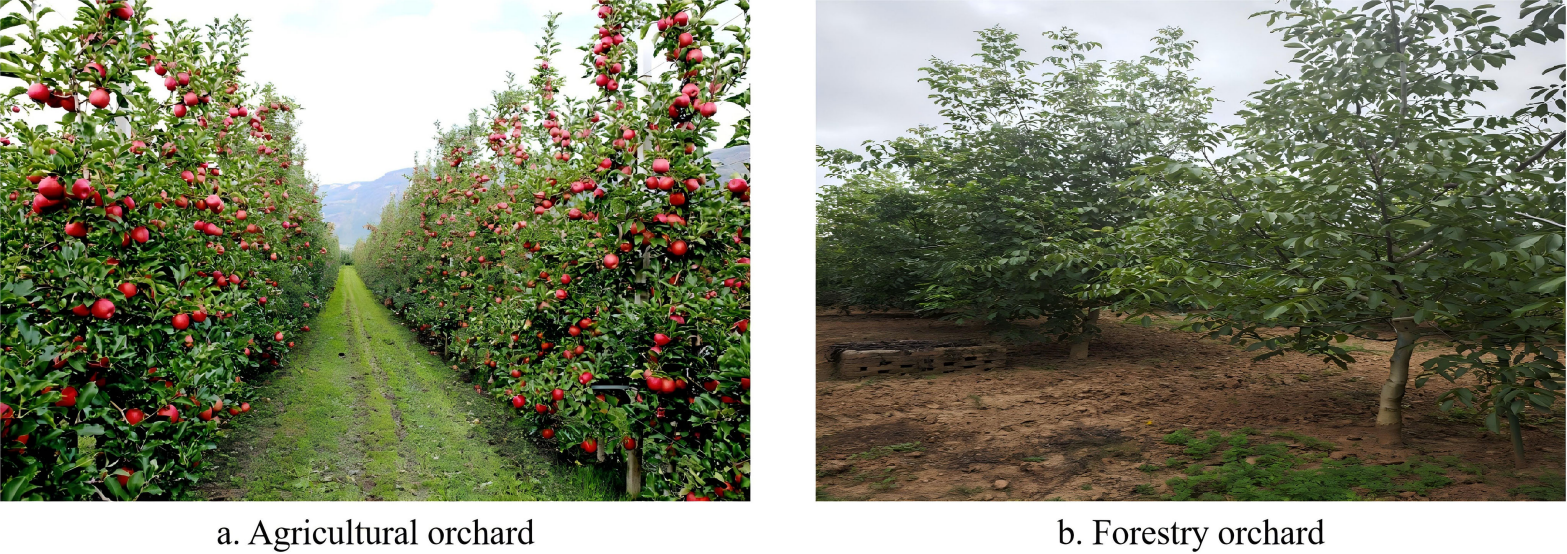
Orchard types. Representative examples of two common orchard planting systems: **(a)** agricultural orchard; **(b)** forestry orchard.

According to the data acquisition approach, modern orchard yield estimation methods are mainly categorized into identification-based estimation and regression-based estimation (Ali and Imran, 2021; Neupane et al., 2023). Identification-based methods typically utilize machine vision to detect and count the number of fruits, and then calculate yield by combining this with the average fruit weight. This approach is suitable for crops such as apples, pears, and grapes, where fruits are prominent and orchards are well managed. However, for trees growing in more complex environments, or with fruits that are easily occluded—such as chestnuts and walnuts—the effectiveness of machine vision methods is limited. In contrast, regression-based methods employ remote sensing technologies like LiDAR and hyperspectral cameras to acquire canopy structure, height, volume, and spectral information, constructing regression models for yield estimation. These methods are more widely used in forestry orchards with complex environments. The differences between agricultural and forestry orchard environments are illustrated in **Figure 1**.

Although machine vision, remote sensing, and deep learning have driven substantial progress in orchard yield estimation, existing studies remain fragmented across crop types, orchard architectures, sensing platforms, and evaluation protocols, which hampers cross-study comparability and complicates method selection for practical deployment (Koirala et al., 2019; Darwin et al., 2021; Maheswari et al., 2021; Jayasinghe et al., 2022). Meanwhile, key deployment challenges—such as fruit occlusion, varying illumination, and data heterogeneity—have yet to be adequately addressed. Therefore, a systematic review grounded in real-world orchard scenarios is needed to consolidate and critically compare the literature, summarize the applicability conditions and trade-offs of different methods and platforms, and synthesize evaluation metrics and experimental settings to support more informed methodological choices and system design for both researchers and practitioners.

This review aims to comprehensively summarize advances in orchard yield estimation using machine vision, spectral remote sensing, and multi-sensor fusion, and to systematically analyze the applicability, strengths, and limitations of different methodological paradigms. In addition, we examine the current status and technical bottlenecks of multi-sensor data fusion for yield prediction in modern orchards, and further discuss emerging trends in intelligent automated operation platforms, identifying key scientific questions that require urgent attention. Ultimately, this work seeks to provide theoretical guidance and technical support for the modernization of the fruit industry. In particular, we establish a unified taxonomy and comparative framework spanning sensing modalities, platform carriers, algorithmic pipelines, and evaluation metrics, with the goal of bridging the gap between methodological advances and scalable, field-level deployment.

## 2 Methodology

**Figure 2:**
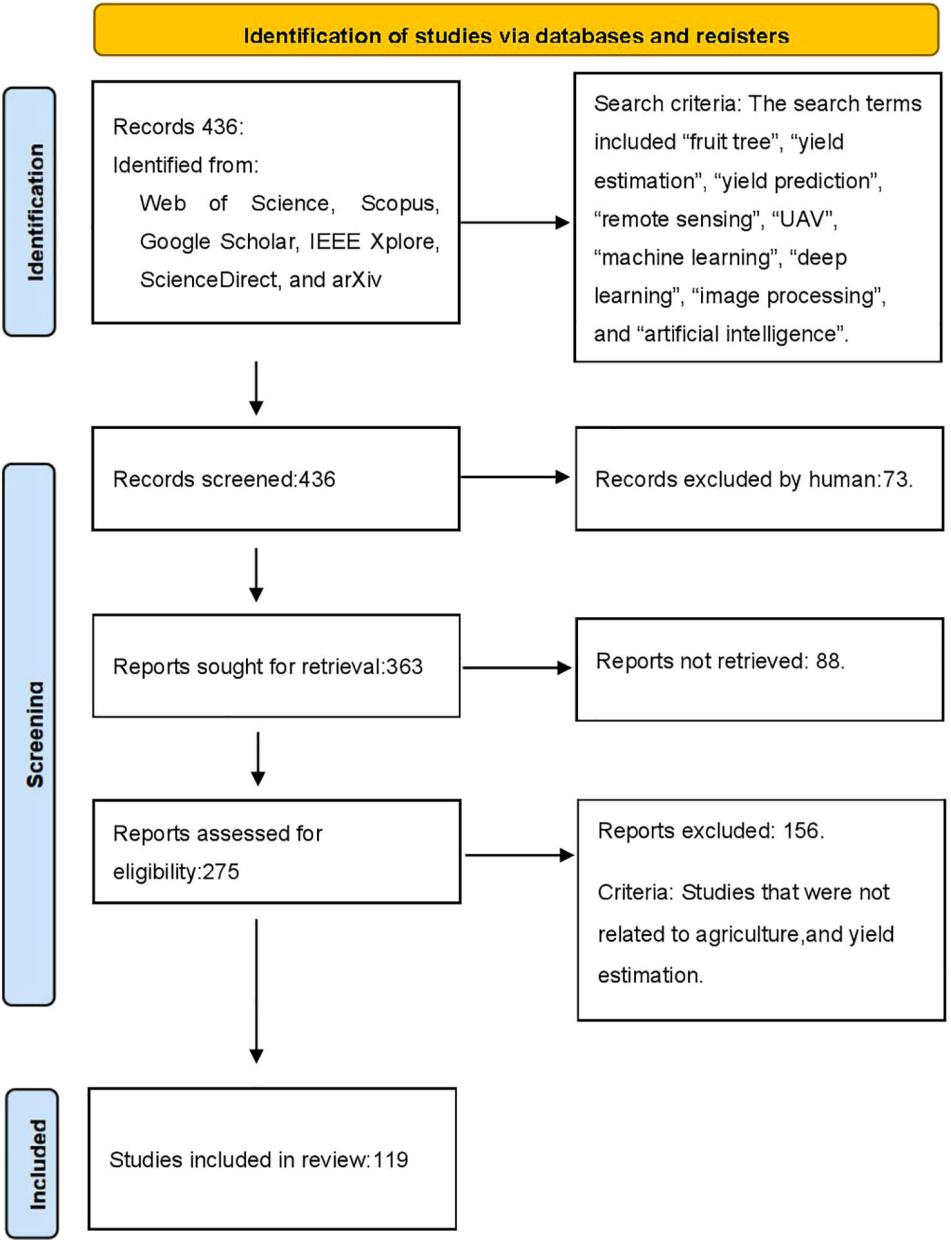
PRISMA 2020 flow diagram for this study, showing the stages of identification, screening, eligibility assessment, and final inclusion, together with the reasons for exclusion at each stage.

This review adopts a systematic approach to comprehensively analyze and evaluate research progress, methodological advantages and disadvantages, and future directions in the field of fruit yield estimation. The process consists of three main stages: literature search, literature screening, and in-depth analysis. First, the research objectives and scope are clarified, focusing on fruit yield estimation technologies for crops such as apple, citrus, and grape. The review covers both traditional manual methods and modern sensor- and AI-based techniques. Comprehensive literature searches are conducted using major academic databases, including Web of Science, Scopus, Google Scholar, IEEE Xplore, ScienceDirect, and SpringerLink. The search strategy employs combinations of Chinese and English keywords, such as “fruit tree,” “yield estimation,” “yield prediction,” “remote sensing,” “UAV,” “machine learning,” “deep learning,” “image processing,” and “artificial intelligence.” The reference lists of selected articles are also explored (snowball method) to further expand the literature coverage (**Figure 2**).

For literature screening, explicit inclusion and exclusion criteria are established. Included studies must focus on fruit yield estimation, be published as peer-reviewed journal articles or high-quality conference papers, and present methodological innovation, practical application, or performance evaluation. The time frame is restricted to the past 15 years to reflect recent advances. Excluded are studies unrelated to fruit crops, lacking practical validation, non-Chinese or non-English publications, studies without accessible full text, or those employing outdated methodologies. An initial pool of approximately 436 records was identified, after which titles/abstracts and full texts were screened.

In the analysis stage, the screened literature is further categorized by technical approach, including traditional methods, image and machine vision methods, remote sensing and UAV techniques, as well as machine learning and deep learning methods. Each category is systematically analyzed for its advantages, limitations, applicability, accuracy, and practical performance. Both qualitative and quantitative comparisons and visual analyses are employed. Finally, the main challenges and limitations of current research are summarized, future research directions are proposed, and the potential and limitations of existing approaches in real-world production are discussed.

## 3 Orchard yield estimation based on machine vision technology

With the continuous advancement of artificial intelligence technologies, machine vision, as an important branch of deep learning, has demonstrated broad application prospects in smart forestry, particularly excelling in key tasks such as fruit recognition and orchard yield estimation (Gongal et al., 2015; Farooqui et al., 2023a). According to different estimation strategies, current methods for orchard yield estimation can be broadly categorized into direct and indirect estimation approaches. In terms of operational procedures, direct yield estimation typically involves two main steps: (1) detecting and counting fruits on individual trees; (2) aggregating the fruit counts from all trees within the orchard to estimate the total yield. Indirect yield estimation, on the other hand, generally comprises three steps: (1) detecting and counting fruits in images; (2) inferring the total number of fruits per tree or tree load using a regression model based on the counted fruits in the images; and (3) combining sample data from all trees in the orchard to ultimately calculate the overall yield. Compared with direct yield estimation, the indirect approach introduces a regression modeling step for extrapolating from local samples to the entire orchard.

Regardless of the estimation strategy, the accuracy of fruit detection and counting remains the core factor affecting yield estimation performance (Saleem et al., 2021). Currently, mainstream fruit identification and counting methods can be divided into two categories: image segmentation-based counting methods and object detection-based counting methods. These two approaches differ significantly in their underlying principles, output results, computational efficiency, and suitable application scenarios. Therefore, this section focuses on these two categories, analyzing their current applications, advantages, and limitations. In addition, this section will further explore the challenges and recent advances in fruitlet and immature fruit recognition, as well as fruit size estimation.

### 3.1 Fruit counting algorithms based on image segmentation

**Figure 3:**
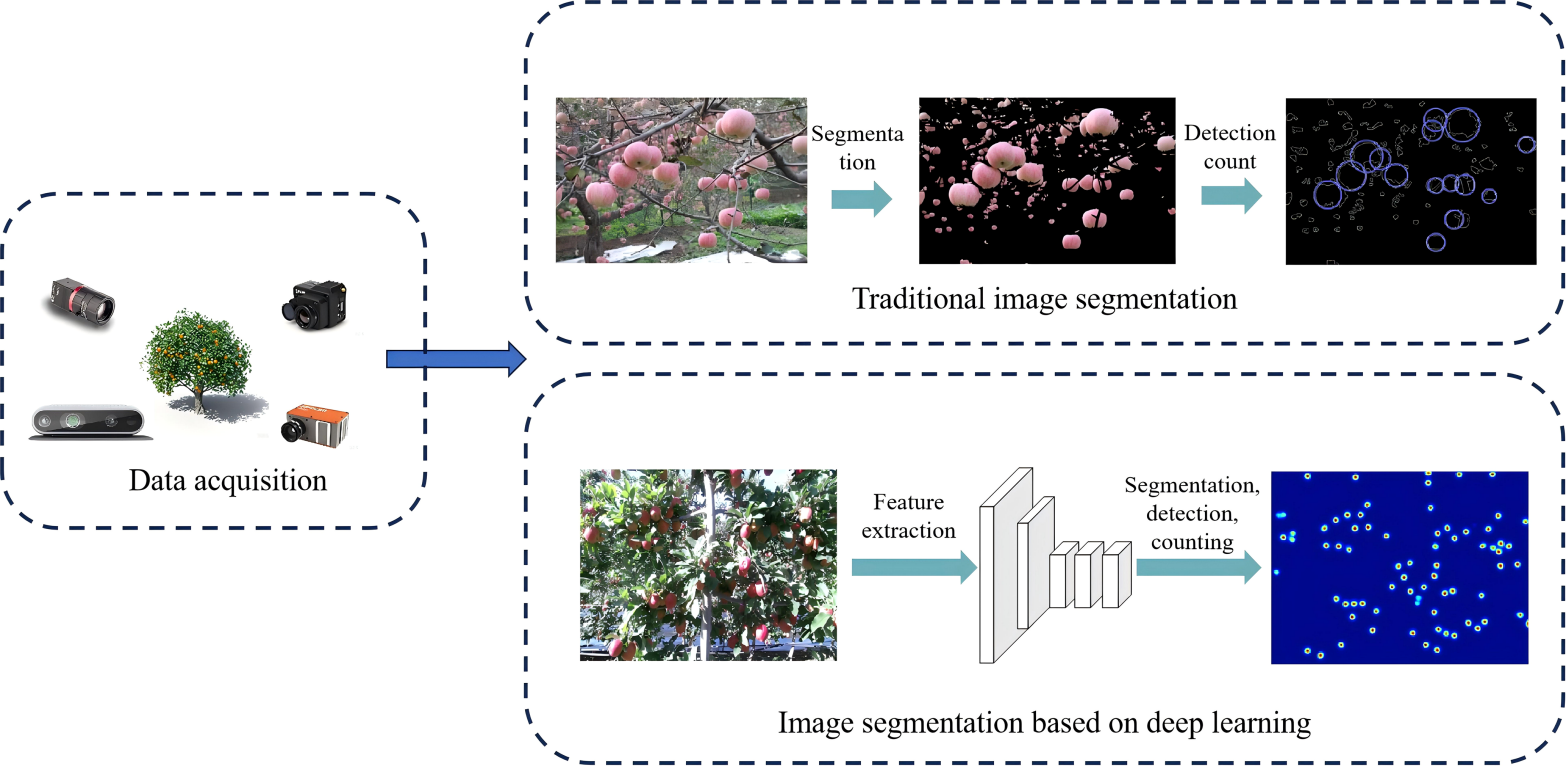
Schematic diagram of threshold segmentation principle. This workflow illustrates typical steps from image acquisition to segmentation (e.g., color/threshold separation), post-processing (e.g., contour extraction/connected-component analysis), and final fruit counting.

Image segmentation is a key research area in computer vision and has been widely applied to fruit identification and counting in orchard yield estimation, forming a fundamental component of precision agriculture. According to algorithmic principles, existing image segmentation methods can be categorized into two main types: traditional threshold-based segmentation and deep learning-based segmentation. The basic workflow of these methods is illustrated in **Figure 3**.

#### 3.1.1 Traditional threshold-based segmentation methods

Traditional image segmentation approaches typically involve two main steps: first, preliminary segmentation of fruit regions is achieved by setting thresholds based on color, texture, or shape features; second, fruit quantity is determined through region feature identification and counting. Hocevar et al. (2014) employed a threshold segmentation method based on the HSL color space, combined with parameter optimization techniques, to enable automatic estimation of apple flower cluster numbers on individual trees, providing effective data support for subsequent yield prediction. To improve segmentation accuracy, some researchers have integrated both color and texture features. For example, Payne et al. (2013) incorporated texture features in addition to color features for fruit segmentation: RGB and YCbCr color spaces were used for initial segmentation, followed by texture analysis to further optimize fruit region identification, and finally, morphological processing was applied for mango fruit counting. Xiao et al. (2015) utilized both color and shape features for apple segmentation: a BP neural network was used to identify apple color features, edge detection was applied to extract shape features, and Hough circle transformation was employed for apple detection and counting, achieving an R² correlation coefficient of up to 0.985 compared with manual counting. Unlike the above studies, which extracted features without prior image preprocessing, Dorj et al. (2017) first converted images to the HSV space and performed noise processing, then segmented citrus fruits based on color features, and subsequently used a distance transform-based watershed algorithm and marker-controlled watershed algorithm for automatic fruit segmentation. The final counting results achieved a correlation coefficient (R²) of 0.93 compared to manual observations.

The above studies utilized RGB images as data sources, with fruit region extraction based on color or texture differences. However, segmentation performance deteriorates significantly when fruits and backgrounds have similar colors or when illumination varies greatly. To address this problem, some studies have introduced hyperspectral imagery, leveraging differences in reflectance between fruits and leaves across different spectral bands to improve segmentation accuracy (Kim et al., 2025).

To compare the performance of different methods for fruit counting tasks, **Table 1** summarizes the research progress of typical threshold-based segmentation methods in terms of feature selection and target adaptability. In summary, traditional threshold-based segmentation methods are simple to implement and computationally efficient, but their reliance on manually set thresholds results in limited robustness and generalizability, making them less effective for fruit segmentation tasks under complex environmental conditions.

**Table 1:** Application of traditional image segmentation algorithms in fruit counting.

Technology	Trait	Goal	Limitation	Reference
Traditional image segmentation	Color/Morphology	Citrus	Sensitive to illumination variations; color features are easily affected by environmental conditions, resulting in low robustness.	Kim et al. (2025)
	Color/Morphology	Apple Blossom	Performs poorly in recognizing fruits under complex backgrounds and occlusions, with limited segmentation accuracy.	Payne et al. (2013)
	Color/Texture	Mango	Struggles to handle reflections and shadow variations on fruit surfaces, leading to restricted texture feature extraction.	Xiao et al. (2015)
	Color/Morphology	Apple	Insensitive to color differences among fruits at different maturity stages, indicating insufficient generalization ability.	Dorj et al. (2017)
	Spectral Reflectance	Citrus	Hyperspectral imaging involves high costs and large data volumes, leading to complex model computation and poor real-time performance.	Bhattarai et al. (2024)

#### 3.1.2 Deep learning-based methods for fruit segmentation and counting

In recent years, deep learning has demonstrated outstanding performance in fruit image segmentation and counting tasks. These methods can be broadly classified into supervised learning and semi-supervised learning approaches. Supervised learning relies on large amounts of labeled data, resulting in high accuracy and strong stability. Semi-supervised learning, on the other hand, leverages a small number of labeled samples alongside a large volume of unlabeled data, reducing the need for extensive annotation and offering greater scalability and cost-effectiveness.

In supervised learning-based research on orchard yield estimation, Bhattarai et al. (2024) proposed the AgRegNet model based on the U-Net architecture to segment flowers and fruits in images and generate density maps (Farooqui et al., 2023b). Yield was then estimated by summing the pixel intensity values in the density maps and constructing a regression model for flower and fruit count estimation. Lin et al. (2024) employed the YOLACT++ instance segmentation model to separate litchi flowers from the background, followed by the FlowrNet algorithm for density map regression, enabling accurate inflorescence counting after segmentation. Ni et al. (2020) used the Mask R-CNN framework for blueberry segmentation, further determining fruit maturity based on color analysis and counting segmented blueberries to estimate yield. Unlike previous studies focused on two-dimensional data segmentation, Gené-Mola et al. (2020) proposed an apple fruit detection method that integrates Mask R-CNN instance segmentation with multi-view imaging. By generating dense point clouds from multi-view images and projecting the two-dimensional detection results into three-dimensional space, the method achieved high-precision fruit localization. Experimental results demonstrated a strong linear correlation between the predicted and actual fruit counts (R² = 0.80).

Supervised learning models typically require manually annotated pixel-level masks for each fruit target, greatly limiting their efficiency in practical applications. To address the heavy reliance on labeled data in supervised learning, Mai et al. (2024) proposed a semi-supervised network called CMCNet for fruit counting. This model estimates density uncertainty via density maps, analyzes the difficulty of fruit pixel classification from a semantic perspective, and visually enhances segmented fruits through colorization, thereby improving the interpretability of visual analysis. **Table 2** summarizes the architectures, experimental performance, and advantages and disadvantages of the aforementioned deep learning models for fruit counting.

**Table 2:** Application of deep learning-based image segmentation algorithms in fruit counting research.

Goal	Model	Model characteristics	Effect	Reference
Apple Blossom and Apple	AgRegNet (based on U-Net)	The model uses point annotation, requiring only one pixel per object, suitable for dense objects and effectively handling occlusion between flowers and fruits.	Lightweight structure with few trainable parameters, inference time is only 14.2 ms.	Bhattarai et al. (2024)
Litchi Flower	FlowerNet (VGG16 backbone with multi-scale feature fusion and density mask generation)	Multi-task learning enables the model to distinguish background and count flowers simultaneously, sharing key info and reducing background interference.	Much larger number of parameters than lightweight models, but smaller counting error.	Lin et al. (2024)
Blueberry	Mask R-CNN	Semi-automatic annotation reduces manual labeling time, improves dataset construction efficiency, and ensures high-quality segmentation labels.	High accuracy, but larger model size and computational complexity, requiring more hardware resources and inference time.	Ni et al. (2020)
Fruit-2019 Dataset	CMCnet (semi-supervised model)	Adaptive thresholding trains difficult pixels first and then easier ones for density map consistency, stabilizing training and increasing efficiency.	Better prediction for small clustered fruits; mixed uncertainty estimation captures small fruit pixels, improving density map accuracy.	Mai et al. (2024)
Fruitlet	YOLOv9-SEG	By integrating a convolutional feature enhancement module with a lightweight attention mechanism, high-precision segmentation can be achieved in complex orchard environments.	It maintains stable recognition performance across different fruit growth stages, offers fast inference speed, and is suitable for embedded deployment.	Sapkota et al. (2025)
Fruitlet	YOLOv8/YOLOv11	The model can accurately detect and perform instance segmentation of immature fruits even under complex conditions with foliage occlusion and varying illumination.	It achieves precise segmentation in highly occluded orchard environments, providing an efficient solution for early fruit recognition and yield estimation.	Sapkota and Karkee (2024)

Compared with traditional methods, deep learning-based image segmentation approaches can automatically extract multi-level image features within an end-to-end training framework, significantly improving segmentation accuracy and model generalization. They also demonstrate greater robustness and adaptability, particularly under non-ideal imaging conditions.

### 3.2 Object detection-based methods for fruit counting

With the increasing complexity of orchard environments and the growing demand for automated yield estimation, research focus has gradually shifted toward object detection methods that offer greater real-time performance and adaptability. Object detection not only enables automatic localization and counting of fruits but also effectively addresses challenges such as fruit overlap and occlusion, making it an increasingly mainstream approach for fruit counting. The performance of object detection methods is primarily influenced by data quality and algorithmic architecture. Therefore, this section provides an analysis of relevant studies from the perspectives of data types and model architectures (Zhang et al., 2020; Safari et al., 2024). In addition, this section will also introduce the emerging research direction of zero-shot object detection, outlining its current development status and key research topics.

#### 3.2.1 Data types for object detection

In orchard yield estimation research, the performance of object detection models is significantly affected by imaging conditions (such as camera angle and ambient lighting) as well as the data format (image or video). This section discusses fruit counting methods based on image and video data, analyzing the impact of imaging conditions and data types on detection performance.

##### 3.2.1.1 Fruit counting based on image data

Image-based data acquisition is widely used in orchard yield estimation, with the core objective of detecting and counting fruits using object detection algorithms. Shooting angle and image quality are critical factors influencing counting accuracy. For example, some studies have employed multi-angle imaging to reduce missed detections caused by fruit occlusion. Mirhaji et al. (2021) utilized the YOLOv4 model to analyze citrus detection performance under various environmental conditions (e.g., cloudy, daytime, and nighttime), finding that detection accuracy improves significantly under sufficient lighting. Additionally, when canopy size is large, multi-view imaging can markedly enhance counting accuracy.

The application of unmanned aerial vehicles (UAVs) has enabled canopy top-view images to become another effective source of image data. Several studies have used UAVs to capture orthophotos of orchard canopies and applied object detection models to count fruits from an overhead perspective, followed by regression analysis for yield prediction. For instance, Arakawa et al. (2024) used UAV aerial images of chestnut tree canopies, counted fruits with the YOLO algorithm, and established linear regression models for yield prediction. Apolo-Apolo et al. (2020) found that yield could be accurately predicted using data from only about 28% of the fruits located at the canopy top. Moreover, the flight altitude of UAVs directly influences image clarity and fruit recognition accuracy; Kwon et al. (2024) systematically studied the impact of different flight heights on citrus fruit counting performance, thereby refining UAV imaging guidelines for yield estimation.

Research on image-based fruit counting demonstrates that multi-view imaging and comprehensive canopy coverage are key to improving counting accuracy. For orchards with large canopy areas, image mosaicking techniques are often used to obtain complete canopy images, reducing errors due to missed or duplicate detections.

##### 3.2.1.2 Fruit counting based on video data

In addition to static images, video-based fruit counting methods have gradually emerged, with the core advantage of leveraging inter-frame object association for target tracking, effectively controlling repeated counting and improving detection accuracy. Compared with static image-based approaches, video counting methods are more complex and typically require greater computational resources for real-time processing.

Current research on video-based counting commonly employs methods such as integrating YOLO models with distance thresholds to filter abnormal fruit detections, utilizing Kalman filters for inter-frame object tracking, and adopting matching algorithms for two-stage detection and tracking. For example, Wu et al. (2023a) proposed an automatic video-based fruit counting algorithm that uses YOLOv4-tiny to detect apples in the background and eliminate abnormal data; Gao et al. (2022) significantly reduced the risk of repeated counting by applying the Kalman filter. Furthermore, advanced inter-frame object tracking algorithms such as cascade matching and SORT have been successfully applied to fruit counting tasks. Wu et al. (2023) further optimized apple counting in videos with a two-stage matching system (detection with YOLO in the first stage, and fruit tracking using a matching algorithm in the second stage). He et al. (2022) used a cascade matching method to associate detection bounding boxes with trajectories and optimize inter-frame matching, effectively avoiding repeated counting. Shen et al. (2023) introduced the SORT algorithm for online multi-object tracking and incorporated counting lines in videos to address the issue of duplicate counts.

In recent years, modern multi-object tracking (MOT) algorithms have been widely integrated into orchard fruit counting pipelines to enhance the consistency of fruit recognition and counting in video sequences. Traditional motion model–based tracking methods, such as Kalman filtering and SORT, offer high real-time performance but often suffer from drift and ID-switch issues in dense canopy scenes with frequent occlusions.

To address these limitations, improved MOT algorithms have been developed and extensively applied to fruit detection and tracking tasks. Tu et al. (2025) proposed a deep learning–based OC-SORT multi-object tracking algorithm combined with YOLOv8 to construct a real-time fruit detection and counting pipeline, effectively mitigating problems of fruit occlusion, target drift, and ID switching in orchard environments. Yang et al. (2024) developed a Multiple Rematching SORT (MR-SORT) tracking framework based on YOLOv8 and BoT-SORT, which improves tracking stability and counting accuracy under complex canopy conditions, providing a new technical pathway for real-time yield estimation in orchards. Similarly, Gené-Mola et al. (2023) demonstrated that the ByteTrack detection-based tracking algorithm achieves higher accuracy and robustness than traditional SORT methods in orchard video-based fruit counting tasks with high visual similarity and severe occlusion, while maintaining real-time performance. Zhai et al. (2025) proposed an improved Kalman filtering algorithm with a dynamic forgetting factor, which, when combined with the YOLOv8n model, significantly enhances the stability and accuracy of multi-object tracking in complex orchard environments.

Although video-based counting methods perform well for fruit trees with prominent features and relatively simple backgrounds (such as apple and citrus trees), they encounter challenges with inter-frame object matching when fruit features are less distinct (as in chestnut or walnut trees), which affects counting accuracy. Future research should focus on solving inter-frame matching and computational efficiency issues in video-based counting, developing more efficient lightweight detection and tracking models to enhance applicability in complex environments. In summary, fruit counting methods based on image and video data each have their own advantages. Researchers should select appropriate counting approaches according to fruit tree species, environmental conditions, and practical requirements, while continuing to explore and improve object detection algorithms to further enhance the accuracy and robustness of orchard yield estimation.

#### 3.2.2 Object detection algorithms

The previous section discussed the impact of data acquisition methods and data types on fruit counting performance. In addition to data quality, the selection and optimization of detection models are also critical factors influencing counting results. Different model architectures exhibit varying performance in terms of detection accuracy and speed. The introduction of specific classification modules can further enhance model detection capabilities. For instance, incorporating multi-scale attention modules enables simultaneous capture of both global features—such as fruit contours and color—and local details, such as fruit texture and peduncles, thereby improving recognition accuracy for fruits of different sizes.

**Figure 4:**
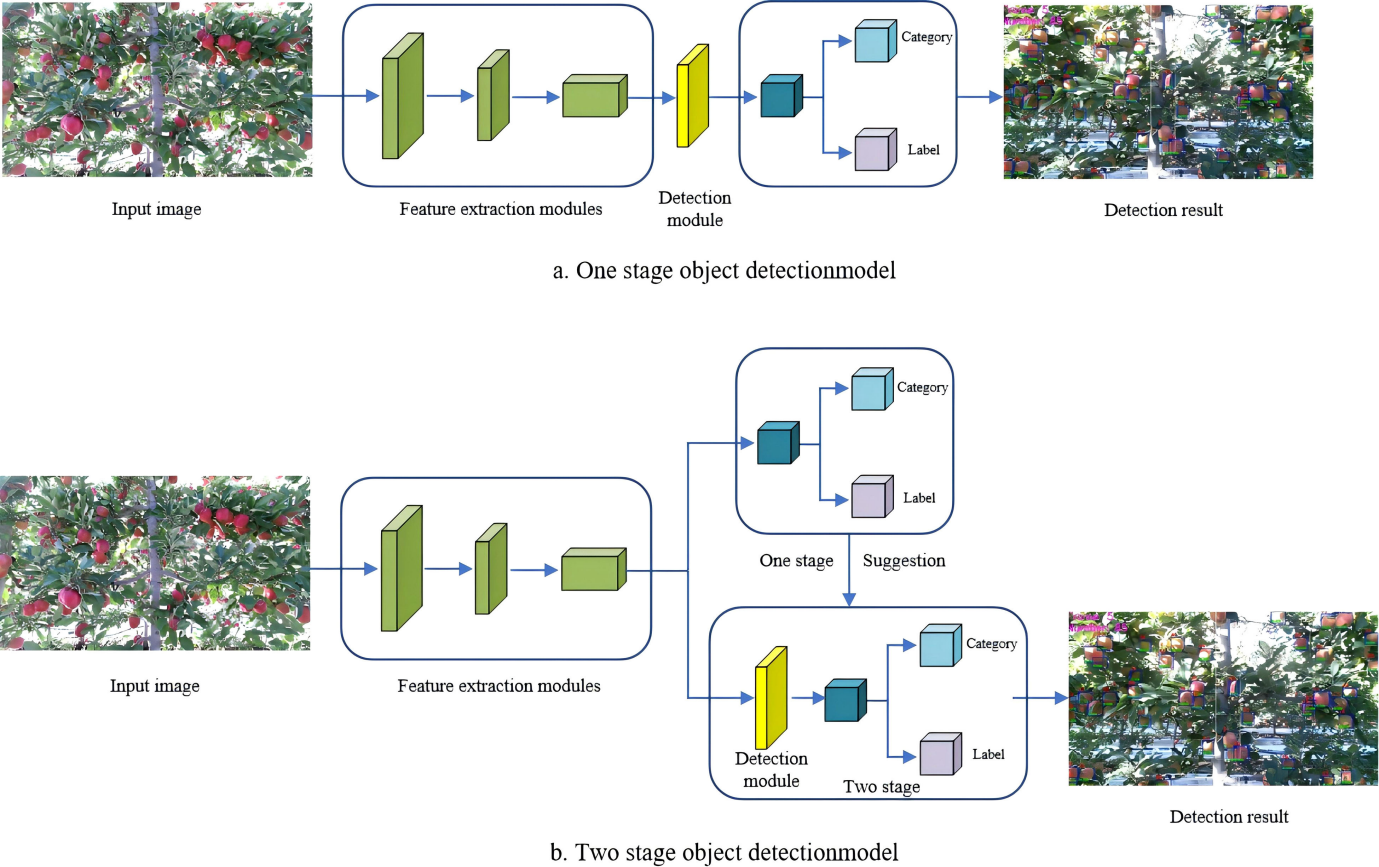
Schematic diagram of object detection model principles: **(a)** one-stage object detection model; **(b)** two-stage object detection model. This diagram conceptually illustrates the general workflow of deep-learning-based object detection, including feature extraction and bounding-box prediction.

Currently, mainstream object detection algorithms are generally divided into two categories: single-stage and two-stage methods. The schematic diagrams of these models are illustrated in **Figure 4**. This section discusses the application and improvement of different types of algorithms in yield estimation. **Table 3** summarizes the characteristics and application scenarios of various object detection algorithms.

**Table 3:** Application of single-stage and two-stage object detection models in fruit counting.

Model type	Data type	Goal	Model and improvement content	Reference
Single-stage model	Image	Citrus fruit	YOLOv4, no improvement	Mirhaji et al. (2021)
	Image	Chestnut	YOLOv4, no improvement	Arakawa et al. (2024)
	Image	Holly fruit	YoloX, no improvement	Zhang et al. (2022b)
	Image	Pomegranate	YOLOv10 refines the backbone and adds attention to speed up inference for real-time use	Liu et al. (2024)
	Image	Yellow peach	Add a CA attention mechanism to the YOLOv5s baseline neck (BiFPN), improving mAP by 6.05% and F1 by 6.72%	Wang et al. (2024)
	Image	Apples, pears	YOLOv7, no improvement	Del Brio et al. (2023)
	Image	Lychee	The backbone network of YOLOv5 has been replaced by a Bidirectional Feature Pyramid Network (BiFPN), improving AP@0.5 from 50.6 to 64.1 (+13.5 pp; +26.7%) over the YOLOv5 baseline.	Xiong et al. (2023)
	Image	Lychee	YOLOv5, no improvement	Peng et al. (2025b)
	Video	Apples	YOLOv4-tiny, no improvement	Wu et al. (2023a)
	Video	Apples	YOLOv4-tiny, no improvement	Gao et al. (2022)
	Video	Apples	YOLOv3, no improvement	He et al. (2022)
	Video	Grapes	YOLOv5, no improvement	Shen et al. (2023)
	Video	Passion fruit	Improve YOLOv5 by replacing its backbone with GhostNet, increasing detection speed by 81%.	Sun et al. (2024)
	Image	Immature fruit	YOLOv8 and Mask R-CNN	Sapkota et al. (2024a)
	Image	Immature fruit	YOLOv8	Sapkota and Karkee (2024)
	Generative Image	Apple	YOLOv11	Sapkota et al. (2024b)
Two-stage model	Image	Citrus fruit	Combine the LSTM model based on Faster R-CNN, reducing yield estimation error from 13.74% to 7.22% (−47.5%) versus the baseline.	Apolo-Apolo et al. (2020)
	Image	Mangoes, pomegranates, apples, oranges	Modify the IoU of the original Faster R-CNN and introduce distance metrics (MIoU), boosting R² from 0.81–0.92 to 0.92–0.98.	Behera et al. (2021)
	Image	Orange	Improve the R-CNN model by adding multilevel training classifiers, increasing F1 from 0.8218 to 0.8678	Mai et al. (2020)
	Image	Pineapple	The Faster R-CNN anchor sizes were optimized and loss normalization was added, achieving an F1-score of 0.849 (IoU > 0.5).	Shiu et al. (2023)

##### 3.2.2.1 Single-stage fruit counting methods

Single-stage object detection algorithms reformulate the detection task as a regression problem, where the network directly predicts object class probabilities and generates bounding boxes. The YOLO (You Only Look Once) series is a representative example of this category, characterized by its high detection speed and simple architecture; it produces predictions in a single model pass, greatly improving fruit detection efficiency. Through multiple generations of development, the YOLO series has evolved to include architectures suited to various requirements, such as lightweight models (focusing on speed) and large-scale models (emphasizing accuracy). Moreover, YOLO features a clear code structure, flexible configuration files, and compatibility with multiple programming languages and platforms, making it easy to modify, optimize, and deploy. As a result, YOLO has become the mainstream object detection algorithm in fruit detection tasks. For example, Mirhaji et al. (2021) applied the classical YOLOv4 model for citrus yield estimation, optimizing the architecture with transfer learning during training to accelerate model convergence and achieving a recognition accuracy of 91.23%. Arakawa et al. (2024) used YOLOv4 to count chestnut fruits on trees, reaching an R² value of 0.98 between the model’s counts and manual counts, demonstrating high-precision chestnut fruit counting. Del Brio et al. (2023) employed a pre-trained YOLOv7 model to detect apples and pears, incorporating fruit size estimation into their study to further improve yield estimation accuracy. Sapkota and Karkee (2024) applied improved YOLOv8 and YOLOv11 models for sour cherry yield estimation, demonstrating robust performance under complex lighting and dense fruit conditions.

While these studies demonstrate the application of YOLO models in fruit detection, most are based on basic YOLO structures without in-depth optimization for the unique characteristics of fruit detection. Recently, many researchers have focused on improving YOLO models for fruit detection and counting tasks. Since the backbone network is responsible for extracting image features and transforming them into semantic feature maps, most improvements target the backbone. The simplest enhancement is the addition of attention mechanisms; for example, Lv et al. (2024) added a CBAM attention module to the PP-YOLOE model to improve detection accuracy, and used local distillation techniques to reduce the model’s parameter count. Beyond simple attention modules, some have replaced the entire backbone to improve performance for fruit detection. Wang et al. (2024) improved the lightweight YOLOv5s by substituting the backbone with a GhostNet architecture integrated with a coordinate attention (CA) mechanism. Zhang et al. (2022b) applied YOLOX for holly fruit detection and classification, incorporating a Focusnet module in the backbone to enhance feature extraction efficiency. Liu et al. (2024) modified YOLOv10 by replacing its original backbone with a VanillaNet architecture and introducing a SimAM attention mechanism to boost feature extraction, achieving accurate pomegranate counting. Xiong et al. (2023) replaced the PANet backbone in YOLOv5 with a BiFPN (bidirectional feature pyramid network) to enhance detection of small litchi fruits.

Beyond backbone improvements, some studies have enhanced the neck (feature fusion part) of the YOLO model to better integrate features. For example, Liu and Yin (2023) embedded a CA attention mechanism into the YOLOv7 backbone to improve yellow peach detection and added a p2 module for downsampling in the neck, thus increasing small object detection accuracy. Peng et al. (2025a) introduced a Transformer structure at the end of the YOLOv5 backbone and added an ECA (Efficient Channel Attention) mechanism in the neck’s C3 module, as well as replacing the activation function to enhance backpropagation efficiency; the improved model increased litchi detection accuracy by 1.5% compared to the original.

##### 3.2.2.2 Two-stage fruit counting methods

Two-stage object detection algorithms typically divide the detection process into two steps: first, generating region proposals, and then refining classification and localization of these regions. Compared with single-stage methods, two-stage algorithms such as Faster R-CNN offer significant advantages in detection accuracy, especially in complex scenarios involving fruit overlap or small object detection. However, they also incur higher computational costs and slower inference speeds. In response to orchard scene complexity, researchers have continuously optimized and extended two-stage detection models to improve detection accuracy and adaptability. Some studies have integrated temporal models, combining Faster R-CNN with deep learning techniques such as LSTM to enable dynamic yield estimation at both the individual tree and whole-orchard scales (Apolo-Apolo et al., 2020). To address challenges such as fruit overlap and small object detection, other studies have introduced distance metrics, adjusted anchor box sizes, or optimized loss function normalization within the model architecture, effectively reducing missed detections and performance drops (Behera et al., 2021; Shiu et al., 2023). Moreover, ensemble approaches—such as combining multiple classifiers—have further enhanced robustness in multi-scale and heterogeneous fruit detection tasks (Mai et al., 2020). Overall, two-stage object detection algorithms are evolving from simple feature extraction and classification to multi-strategy integration and intelligent adaptation, driving improvements in yield estimation performance under complex real-world conditions.

Despite their superior detection accuracy, two-stage models are generally more computationally intensive and slower in inference, and their generalizability is relatively weaker in natural orchard environments, where uneven lighting, fruit occlusion, and background interference are common. As a result, the actual application of two-stage models in fruit detection and counting remains limited. By contrast, single-stage algorithms such as YOLO offer significant advantages in detection speed and deployment efficiency, and can meet the basic accuracy requirements for non-occluded fruit scenarios. Therefore, single-stage models remain the mainstream choice for fruit counting, particularly in agricultural production scenarios where real-time performance and computational resources are critical.

Semantic segmentation and object detection each have their own strengths and limitations. Comparative analysis reveals that although semantic segmentation entails higher annotation costs and training overhead, it offers significant advantages in providing fine-grained detection results. In contrast, object detection methods have simpler architectures and are more suitable for less complex scenarios. To integrate the advantages of both approaches, Liu et al. (2022) developed a hybrid model combining object detection and semantic segmentation. The first stage uses object detection to locate the fruit, while the second stage employs instance segmentation to refine pixel-level information in local regions. Although a number of such hybrid models have been proposed, their application in fruit detection remains limited, and their effectiveness in fruit counting tasks has yet to be fully explored.

#### 3.2.3 Detection of small sample and zero sample fruits

In recent years, with the widespread application of deep learning models in object detection tasks, the dependence of such models on large-scale annotated datasets has become increasingly prominent. However, in the field of orchard yield estimation, the diversity of fruit species, variations in morphology, and the complexity of acquisition environments make the construction of comprehensive and high-quality datasets highly costly. This has greatly limited the generalization ability of conventional supervised detection models. To address this issue, Few-Shot Learning (FSL) and Zero-Shot Learning (ZSL) have gradually emerged as important research directions for fruit object detection.

##### 3.2.3.1 Few-shot fruit object detection

Few-shot learning aims to recognize and detect new object categories using only a small number of labeled samples. Its core idea is to fully train a model on base categories and then rapidly adapt it to novel categories through feature transfer or meta-learning strategies. In fruit detection scenarios, different fruit tree species exhibit significant variations in morphology but share certain similarities in structural and textural characteristics. Therefore, few-shot learning enables effective cross-species detection under limited data conditions.

For example, James et al. (2024) achieved high-precision fruit segmentation under few-shot conditions by performing specialized transfer pretraining on a citrus dataset and fine-tuning the model on an apple dataset. Similarly, James et al. (2024) fine-tuned a RetinaNet model under few-shot settings to achieve accurate fruit object detection. The authors adopted a transfer learning strategy, using pretrained features from general datasets as the foundation and fine-tuning them with a small number of fruit samples, thereby enabling the model to quickly adapt to new fruit categories. The application of few-shot learning provides new opportunities for multi-species orchard yield estimation, especially in scenarios with limited samples or newly introduced fruit varieties.

##### 3.2.3.2 Zero-shot fruit object detection

Unlike few-shot learning, zero-shot learning (ZSL) aims to detect novel object categories without using any annotated samples. Its core concept lies in establishing a mapping between visual and semantic features through semantic embeddings (e.g., word vectors or textual descriptions), thereby enabling knowledge transfer “from the known to the unknown.” In recent years, vision-language alignment models such as Grounding DINO have demonstrated outstanding performance in open-vocabulary object detection tasks.

In the context of orchard yield estimation, zero-shot detection offers an effective solution to the challenges of recognizing diverse fruit species and the scarcity of annotated data. By leveraging textual prompts (e.g., “red round fruit” or “fruit with spiny husk”) aligned with image features, models can automatically detect unseen fruit categories. For instance, detection frameworks based on Grounding DINO enable unified detection across multiple fruit tree species without requiring individual annotation and training for each fruit type, significantly reducing dataset construction costs (Mullins et al., 2024; Singh et al., 2025; Zhu et al., 2025).

Beyond directly applying the Grounding DINO architecture for fruit detection, several studies have further integrated domain-specific multimodal representation models into the framework by embedding or distilling features from CLIP or its agriculture-oriented variant AgriCLIP (Nawaz et al., 2024). AgriCLIP is pretrained on large-scale agricultural image–text corpora to construct a cross-modal representation space that better aligns with crop and pest semantics. This fusion strategy enhances the model’s semantic perception of fruit species, maturity levels, and complex environmental conditions, thereby significantly improving the accuracy and generalization performance of zero-shot detection in orchard scenarios.

### 3.3 Fruitlet detection and recognition

Fruitlet recognition is a critical component of orchard yield estimation and precision cultivation management, playing an essential role in early fruit set assessment, intelligent fruit thinning, and growth monitoring. The fruitlet stage represents a key physiological phase in the transition from flower to fruit, during which the quantity and spatial distribution of fruitlets directly determine subsequent fruit competition, physiological fruit drop rate, and potential yield level.

Compared with mature fruit detection, fruitlet recognition poses greater technical challenges — fruitlets are typically small in size, exhibit color similarity to surrounding foliage, have unstable morphological characteristics, and are often subject to occlusion by branches and leaves or affected by illumination variations. Therefore, developing models capable of reliably recognizing fruitlets under complex natural orchard conditions is a prerequisite for achieving automated early-stage yield estimation and dynamic monitoring.

For instance, Sapkota et al. (2024b) employed the YOLOv8 model for branch and immature fruit segmentation in complex apple orchard environments. By training the model on both dormant- and growing-season imagery, it achieved high detection accuracy and real-time performance even under challenging conditions with heavy foliage occlusion and varying illumination. Furthermore, Sapkota and Karkee (2024) combined YOLOv8 and YOLOv11 models to perform instance segmentation of both occluded and non-occluded immature green apples in commercial apple orchards in Washington State, systematically evaluating model robustness under diverse environmental conditions. The results demonstrated that YOLO-based models can achieve precise fruitlet recognition and segmentation even in highly occluded scenarios, providing an efficient and reliable solution for early-stage fruit detection and yield estimation in orchard environments.

### 3.4 Fruit size estimation

Fruit size is one of the most critical parameters in orchard yield estimation, as it directly determines the weight of individual fruits and ultimately affects the accuracy of total yield prediction. Unlike detection methods that merely count the number of fruits, fruit size estimation combines geometric information—such as diameter, area, or volume—with average density or empirical weight coefficients, thereby enabling the transition from qualitative recognition to quantitative yield estimation. With the rapid advancement of 3D imaging, multi-view reconstruction, and depth sensing technologies, fruit size estimation has evolved from traditional two-dimensional (2D) measurement to high-precision three-dimensional (3D) analysis.

#### 3.4.1 Size estimation based on 2D images

Early studies mainly relied on 2D image analysis, inferring fruit diameter or area through pixel measurements. These methods typically calibrated the imaging system using a reference board or known scale parameters and then estimated fruit size through geometric projection models. For example, Orly Enrique Apolo-Apolo et al. (2020) utilized known-size rulers placed near trees in unmanned aerial vehicle (UAV) imagery to establish a conversion ratio between pixel and actual length. After detecting fruits, the pixel area of each fruit was converted to its actual diameter under the assumption of a circular shape, thereby achieving fruit size estimation. Lu et al. (2022) proposed an early apple load estimation method based on Canopy-Attention-YOLOv4 (CA-YOLOv4), which further enabled fruit size estimation after detection. In their study, a reference fruit of known dimensions (80 mm × 90 mm) was placed in each canopy image, and the actual height and width of fruits were calculated using the pixel ratio between detected bounding boxes and the reference fruit. The results indicated that the method achieved high estimation accuracy even without depth data (R² ≈ 0.65–0.68, RMSE ≈ 10–11 mm), providing a feasible approach for low-cost and rapid early crop load estimation. However, despite their simplicity and computational efficiency, 2D methods are highly sensitive to camera angle, illumination variation, and fruit occlusion, often leading to underestimation or misjudgment of fruit size under natural orchard conditions.

#### 3.4.2 Size and volume estimation based on 3D reconstruction

To overcome the limitations of two-dimensional imaging, an increasing number of studies in recent years have adopted stereo vision and structure-from-motion (SfM)-based reconstruction methods to obtain spatial point clouds of fruits, enabling more accurate measurement of fruit volume and morphology. Gené-Mola et al. (2021) combined Mask R-CNN instance segmentation with SfM photogrammetry for apple yield estimation, achieving an RMSE of 6.42% and an R² of 0.80 between the predicted and actual fruit counts. This approach not only improved detection accuracy but also enabled three-dimensional localization and volumetric measurement of individual fruits. Masoudi et al. (2024) proposed an SfM-based fruit surface modeling approach designed to address the difficulty of feature matching on smooth and reflective fruit surfaces. By applying a micro-particle coating layer to the surface of tomatoes, the method substantially enhanced the quality of SfM reconstruction, reducing the estimation errors of volume, surface area, and color index to 2.3, 2.4, and 0.35 RMSE, respectively, with an overall error of less than 5%.

Beyond SfM-based techniques, the emerging Neural Radiance Field (NeRF) technology has provided a new solution for 3D reconstruction in orchard environments. Unlike SfM, NeRF does not rely on explicit feature matching but instead reconstructs real-world orchard characteristics through volumetric rendering in an implicit space, thereby improving reconstruction stability under uneven lighting, fruit reflectivity, and partial occlusion. For instance, Peng et al. (2025a) developed a UAVO-NeRF model that integrates UAV multi-view imagery with hash-encoding acceleration, achieving high-precision 3D reconstruction and semantic segmentation of orchard rows. Furthermore, FruitNeRF and FruitNeRF++ combined the NeRF framework with instance-level semantic segmentation, enabling direct 3D localization and counting of fruits. Although NeRF has not yet been widely applied to precise fruit size or volume estimation, its integration with depth information and semantic constraints holds great potential for achieving accurate 3D fruit measurement and yield estimation in orchard scenarios.

#### 3.4.3 Size estimation based on depth sensing and multimodal imaging

To address the planar limitations of 2D methods and the computational complexity of 3D reconstruction, recent research has explored depth-sensing (RGB-D) and multimodal imaging strategies for fruit size estimation. These approaches employ structured light, time-of-flight (ToF), stereo vision, or multi-sensor fusion techniques integrating infrared and laser modalities to directly acquire depth and shape information, which are then processed by deep learning models for diameter and volume prediction. For example, beyond simple two-dimensional size estimation methods that rely on auxiliary reference tools within images, some researchers have utilized depth cameras to quantify the physical dimensions corresponding to 2D detection bounding boxes, thereby enabling final yield estimation (Checola et al., 2025). Overall, depth-sensing and multimodal imaging approaches offer a balance between accuracy and efficiency, reducing sensitivity to environmental factors while enabling more reliable and automated fruit size estimation in real-world orchard conditions.

## 4 Orchard yield prediction based on spectral remote sensing

Orchard yield estimation methods based on machine vision typically focus on the phenotypic characteristics of fruit trees—such as fruit count and flower number—and infer final yield through statistical analysis of these features. However, fruit yield is also influenced by internal factors such as genetics and physiology. Consequently, in addition to estimating yield by identifying and counting phenotypic features, an increasing number of researchers are employing spectral remote sensing techniques to retrieve physiological and ecological characteristics at the canopy scale. This approach enables spatiotemporal monitoring of tree health and physiological dynamics, providing valuable support for more comprehensive and accurate yield prediction.

Due to the complexity of orchard environments, there are stringent requirements on the accessibility of remote sensing platforms. As a result, ground-based remote sensing is relatively limited in orchard yield estimation applications. Most remote sensing sensors used for yield estimation are mounted on UAV (unmanned aerial vehicle) or satellite platforms. According to monitoring scale, remote sensing applications can be categorized into field-scale and regional-scale monitoring, each suited to different application scenarios. This section discusses the latest advances and practical applications of remote sensing platforms at various scales in orchard yield estimation.

### 4.1 Field-scale yield estimation

Field-scale orchard yield estimation primarily targets small, well-defined orchards and typically utilizes UAVs (unmanned aerial vehicles) as sensor platforms for data acquisition. UAV remote sensing data offer high timeliness and spatial resolution, and the data collection process is not constrained by revisit cycles. Currently, there are two main categories of UAV-based remote sensing approaches for orchard yield estimation. The first category relies on the spectral reflectance differences among various tree canopy components to segment fruit pixels, followed by yield estimation using regression methods. The second category extracts the spectral reflectance of the entire canopy and builds regression models based on the nonlinear relationship between spectral features and single-tree fruit weight to estimate yield.

At present, research on the first category is relatively limited, with only a few case studies. For example, Zhou et al. (2023) used the Color Change Vegetation Index (CCVI) to segment dragon fruit from the background, enabling yield estimation for individual trees. In the second category, empirical models based on vegetation indices are among the mainstream methods. Niu et al. (2023) estimated pomegranate yield using multiple vegetation indices—including the Normalized Difference Vegetation Index (NDVI), Green Normalized Difference Vegetation Index (GNDVI), and Normalized Difference Vegetation Index of Red Edge (NDVIre)—as well as canopy area features, combined with machine learning algorithms. Some researchers have further improved the quality and accuracy of UAV remote sensing data through image processing. For instance, Fan et al. (2024) applied multispectral image super-resolution reconstruction techniques to enhance image quality, and then extracted spectral indices from the reconstructed images to improve the accuracy of ginkgo yield prediction.

In addition, some studies have explored the relationship between remote sensing monitoring time points and fruit yield. As perennial woody plants, fruit trees may be affected by external factors during specific periods of the growing season, which can ultimately influence yield. Therefore, some researchers have begun to examine the relationship between spectral data from different phenological stages and yield variables. For example, Van Beek et al. (2015) collected multispectral canopy data of pear trees at different growth stages and analyzed the relationships between various spectral indices and fruit yield, enabling indirect estimation of pear yield. Carrillo et al. (2016) used multispectral canopy data from grapevines over different years and estimated grape weight using a target sampling approach based on NDVI. Zhang et al. (2025) combined UAV multispectral data and multiple machine learning models to estimate individual apple yields at different growth stages, proposing a growth stage stacking ensemble (GSSE) approach. The GSSE model significantly improved estimation accuracy and enabled precise orchard-scale yield mapping.

Overall, existing studies demonstrate the significant application potential of UAV-based remote sensing technology for field-scale orchard yield prediction. Current research mainly focuses on multispectral remote sensing, which offers clear advantages in spatial resolution and flexible data acquisition, providing an important data foundation for yield estimation. With the development of sensor technology, higher-quality data—combined with information on topography, climate, and other factors—will enable high-precision field-scale yield prediction.

### 4.2 Regional-scale yield estimation

Orchards are often distributed in mountainous areas, characterized by complex terrain and scattered plots. Field-scale spectral data acquisition devices face limitations in speed and convenience when applied to large-scale yield estimation. In recent years, with rapid advances in aerospace technology, the revisit cycle of satellite remote sensing has been significantly shortened and the quality of sensor imaging has greatly improved, resulting in an increasing number of studies utilizing satellite remote sensing for forestry resource monitoring and providing essential data support for orchard yield estimation. For example, Shi et al. (2022) used Sentinel-2 satellite remote sensing data to predict goji berry yields across different seasons, although a considerable deviation remained between predicted and actual yields. Yadav et al. (2002) employed IPS satellite data to estimate mango orchard planting area and yield, which enabled rapid yield estimation but with limited predictive accuracy.

Compared with low-resolution satellite remote sensing, the continuous upgrades of sensors on commercial satellites in recent years have greatly improved image resolution. For instance, commercial satellites such as WorldView and SPOT now provide imagery with resolutions as fine as 0.3 meters, sufficient to meet the requirements of individual tree detection. In terms of practical applications, Tanveer et al. (2024) utilized high-resolution spectral images of mango orchards acquired by Landsat-8 to ultimately achieve yield prediction for 2,150 individual mango trees. Rahman et al. (2018) estimated mango yield using high-resolution satellite imagery from WorldView-3, constructing yield regression models by selecting multiple spectral indices and canopy area as independent variables.

High-resolution satellite remote sensing imagery not only enables accurate localization of individual fruit trees but also provides high-quality spectral information, laying a solid data foundation for fine-scale yield estimation at the single-tree level. Similar to multi-temporal UAV remote sensing at the field scale, regional-scale satellite remote sensing applications also rely heavily on multi-temporal data to improve prediction accuracy and stability. Recent studies have increasingly revealed the close relationship between the remote sensing spectral characteristics of fruit trees at different phenological stages and final yield. For example, the application of multi-temporal remote sensing imagery can capture spectral changes during key growth stages such as flowering, shoot elongation, and fruit expansion, and can dynamically reflect tree growth and yield potential using vegetation indices (e.g., NDVI, EVI) (Sun et al., 2017; Rahman et al., 2022). Numerous empirical studies have shown that remote sensing indicators from different phenological stages vary in their predictive power for fruit yield, with spectral parameters during flowering and growth stages generally exhibiting higher yield sensitivity (Bai et al., 2019, Bai et al., 2021). Additionally, yield estimation models developed from multi-year and multi-variety time-series remote sensing data further enhance the accuracy and generalizability of regional-scale orchard yield prediction (Kamangir et al., 2024). Collectively, these findings demonstrate the significant complementarity and application potential of satellite and UAV remote sensing for time-series monitoring and large-scale yield estimation.

Studies on fruit yield estimation using multi-temporal remote sensing have shown that the canopy spectral reflectance during the growing season exhibits the strongest predictive power for final yield assessment. The data acquisition timing and specific targets for multi-scale, time-series remote sensing-based yield estimation are summarized in **Table 4**. However, in actual production, fruit yield is influenced by various factors such as climate and topography. For instance, frost events during the flowering period can affect pollination and fertilization, resulting in a reduced fruit set rate. Therefore, relying solely on spectral reflectance for yield estimation may lead to data source limitations and affect model stability. Torgbor et al. (2023) achieved mango yield estimation by integrating time-series remote sensing data with seasonal weather variables, and their results demonstrated that models combining weather and canopy remote sensing data outperformed single-factor models in prediction accuracy. In addition to environmental factors, Zhang et al. (2019) incorporated historical yield data into a spectral data-based model for almond yield estimation, and found that including yield data from the previous two years as input variables significantly improved yield prediction accuracy. Torgbor et al. (2024) conducted their study using multi-temporal high-resolution satellite imagery and random forest models to automatically predict mango yield at both the individual tree and block scales. They found that the relationship between vegetation indices and yield varied significantly across cultivars, regions, and years, but high-accuracy yield estimation could still be achieved at the block scale.

**Table 4:** UAV and satellite remote sensing time-series data and crop types.

Platform	Time series data	Crop name	Reference
Satellite	Flowering period, new bud growth cessation period, autumn bud growth period, autumn bud growth cessation period	Apple	Rahman et al. (2018)
Satellite	The cumulative time series data of 120 different time interval days during the harvest period from early March to early September	Grapes	Sun et al. (2017)
Satellite	Flowering, vegetation growth, fruit ripening, and harvest	Avocado	Rahman et al. (2022)
Satellite	Time series data from April to July	Grapes	Kamangir et al. (2024)
drone	Young fruit stage, late fruit drop stage, fruit maturity stage, harvest stage	Pear	Van Beek et al. (2015)
drone	Early June, early July, early August, late August	Grapes	Kasimati et al. (2023)

In summary, whether based on satellite or UAV remote sensing, the use of multi-temporal imagery and key parameters such as various vegetation indices enables effective estimation of fruit yield. In specific studies, it is essential to select appropriate sensor platforms according to different tasks in order to improve the specificity and accuracy of remote sensing-based yield estimation for fruit trees.

## 5 Orchard yield estimation based on data fusion

Currently, single-sensor approaches in orchard yield estimation are limited in the dimensionality of information they provide, making it difficult to comprehensively reflect the actual state of fruit trees under complex environmental conditions and ultimately affecting the accuracy and robustness of yield estimation. For example, fruit counting methods based on machine vision are susceptible to occlusion and viewpoint limitations, often resulting in missed detections, while reflectance regression methods based on spectral remote sensing mainly capture canopy physiological information but lack direct characterization of fruit quantity and distribution. To address these limitations, multi-sensor data fusion technology has gradually emerged as an important approach for improving the accuracy of yield estimation (Khaleghi et al., 2013; Chlingaryan et al., 2018). According to the level of fusion and information complexity, existing data fusion methods can be classified into three categories: data-level, feature-level, and decision-level fusion. While a certain degree of experience with multi-source data fusion has been accumulated in the field of crop yield estimation, the application of multi-source fusion in orchard yield estimation remains in the exploratory stage (Ouhami et al., 2021; Ahmad et al., 2022; Islam et al., 2024).

### 5.1 Data-level fusion

Data-level fusion technology integrates raw data of the same type collected from multiple sensors, aiming to maximize the retention of original information. The advantage of this fusion strategy is that it can effectively enhance the quality of information from a single data source—for example, by improving image clarity, optimizing feature representation, or expanding coverage—thus improving the accuracy of subsequent object detection and yield estimation. However, data-level fusion typically requires rigorous data synchronization, spatial registration, and noise control, imposing high demands on computational resources and processing algorithms, and limiting its applicability in cross-modal data analysis.

In orchard yield estimation, typical applications of data-level fusion include image mosaicking and pixel-level fusion. For example, Zhang et al. (2022a) used multi-view image mosaicking to obtain a complete panorama of the tree canopy, reducing fruit occlusion from single viewpoints and thereby improving counting accuracy. In addition, pixel-level fusion involves weighted integration of pixel data from multiple sensors or under different imaging conditions, which comprehensively optimizes the color, texture, and shape features of target regions and helps to reduce detection errors. Such approaches have been more widely applied in crop yield estimation; for instance, Zhou et al. (2018) reconstructed pixel features of target areas using weighted statistical methods, effectively improving the accuracy of wheat spike counting.

In remote sensing-based yield estimation, data-level fusion has demonstrated great potential and application prospects. While satellite remote sensing data offer significant advantages in coverage and continuous monitoring capability, their spatial resolution and susceptibility to weather conditions limit their ability to achieve precise yield estimation at the individual tree level (Yuan et al., 2024). To overcome these limitations, researchers have fused high-resolution UAV remote sensing data with satellite data. This multi-scale fusion not only improves spatial accuracy but also expands the observation range. Currently, the fusion of data from different remote sensing platforms has not been widely applied to orchard yield estimation, but has seen more extensive application in crop yield prediction. For example, the integration of UAV and Sentinel-2 satellite data has enabled successful multi-scale spatial distribution prediction of wheat yield (Anderegg et al., 2024). Other researchers have proposed edge pixel alignment algorithms to effectively fuse Sentinel-2 and PlanetScope satellite data, significantly improving the accuracy of regional-scale crop yield estimation (Amankulova et al., 2024).

At present, data-level fusion techniques are still applied relatively infrequently in fruit yield estimation, although there is considerably more practice in crop yield estimation. Despite some progress, significant challenges and bottlenecks remain for its practical implementation. On the one hand, data-level fusion places high demands on data quality, sensor synchronization, and image registration, which are often difficult to satisfy in real-world orchards with complex environments and diverse data sources. On the other hand, data-level fusion is unable to effectively integrate cross-modal information, which limits its ability to fully leverage the advantages of multi-source heterogeneous data—an issue that becomes even more pronounced in complex orchard environments or when spatial scales differ substantially. Therefore, the practical application of data-level fusion in orchard yield estimation still requires further improvement.

### 5.2 Feature-level fusion

Feature-level fusion focuses on the extraction, transformation, and integration of highly representative features from different sensor data, enabling effective expression and fusion of multi-source features through advanced algorithms. Compared to data-level fusion, feature-level fusion can more effectively eliminate redundant information and noise, enhance data generalizability, and is better suited for in-depth analysis of cross-modal and multi-modal data.

In the field of crop and orchard yield estimation, research on feature-level fusion was initially based on traditional machine learning approaches. Researchers typically integrated texture features from RGB images and spectral features from multispectral data to construct more discriminative feature sets. For example, some studies have fused RGB and multispectral data to support accurate prediction of maize leaf area index (LAI), demonstrating that feature fusion methods significantly improve prediction accuracy compared with single data sources (Ma et al., 2023). In another example, research on faba bean yield estimation constructed feature sets from RGB and multispectral data and applied various machine learning models such as Support Vector Machines (SVM), Ridge Regression (RR), Partial Least Squares (PLS), and K-Nearest Neighbors (KNN). The results showed that multi-source data fusion can substantially enhance yield estimation performance (Cui et al., 2023).

Moreover, feature fusion techniques have gradually extended to the fusion of cross-platform data. Researchers have used methods such as Principal Component Analysis (PCA) to combine satellite and UAV data, improving spatial resolution while preserving original spectral information, thereby enhancing overall agricultural monitoring capabilities (Jenerowicz and Woroszkiewicz, 2016). Some studies have further explored the fusion of UAV and satellite remote sensing data, with experiments showing that feature fusion of high-resolution satellite data and UAV data can more effectively support regional-scale crop yield estimation (Marzougui et al., 2023).

However, traditional machine learning methods face limitations in feature extraction capability and generalizability in complex and dynamic orchard environments. In recent years, the rise of deep learning has introduced new approaches and more powerful support for feature fusion. Deep learning’s ability for automated feature extraction and end-to-end learning has greatly improved the efficiency and accuracy of feature-level fusion. For example, the Deep4Fusion model, developed based on deep learning, achieved an over 20% improvement in forage yield estimation accuracy through automatic feature extraction and fusion (Rodrigues et al., 2023). Additionally, new deep fusion models based on U-Net and Transformer architectures have emerged in recent years, demonstrating significant performance advantages in fusing RGB images and spectral data and surpassing the limitations of traditional methods.

Feature-level fusion is widely applied in agricultural yield estimation, with most related research focused on traditional field crops. On the one hand, the targets of fusion have gradually expanded from multi-sensor integration within a single platform to multi-sensor integration across multiple platforms, achieving richer and more diverse information integration and improving the spatial adaptability and generalization of yield estimation. On the other hand, fusion strategies have evolved from traditional machine learning approaches to deep learning methods. However, applications of feature-level fusion in orchard yield estimation remain relatively limited, and research in this area is still in its early stages. Therefore, there remains significant potential for further development of feature-level fusion in orchard yield estimation.

### 5.3 Decision-level fusion

Decision-level fusion primarily integrates the outputs of predictive models independently established from different sensors or data sources, achieving comprehensive decision-making at a higher level. In contrast to data-level and feature-level fusion—which only integrate local information at the data or feature stages—decision-level fusion can effectively combine the overall predictive strengths of multi-modal and multi-scale data, thereby significantly enhancing the overall accuracy and robustness of yield estimation models. This approach is particularly suitable for orchard management scenarios characterized by diverse data sources and highly variable environmental factors.

In recent years, decision-level fusion techniques have demonstrated promising application effects in orchard yield prediction. For example, some researchers have fused remote sensing data with meteorological variables (such as diurnal temperature range and minimum temperature) to construct models that predict the impact of frost on apple yield (Zhu et al., 2021). In addition, there are studies that combine UAV remote sensing data with long short-term memory (LSTM) temporal models to accurately predict citrus yield under multiple influencing factors (Apolo-Apolo et al., 2020). Other researchers have fused tree water content data with remote sensing spectral data to effectively predict yield variation in olive and peach trees (Sepulcre-Canto et al., 2007).

For orchard yield estimation in complex environments, integrating multi-source sensor data—such as LiDAR, UAV multispectral data, and vegetation indices—with advanced machine learning models (e.g., ensemble learning methods) has successfully enabled accurate yield estimation for apples and other fruit crops (Chen et al., 2022). Some researchers have also collected a variety of morphological trait data, selected seven key features through principal component analysis (PCA), and used these as input variables to construct artificial neural network (ANN) and multiple linear regression (MLR) models for predicting the yield of individual apple trees (Bharti et al., 2023). Additional studies have combined remote sensing and meteorological data (such as NDVI maps with rainfall and temperature data) to comprehensively monitor the growth processes of crops such as grapes, laying the foundation for further improvements in yield prediction accuracy (Kasimati et al., 2023).

Despite the advantages of decision-level fusion methods, their application still faces several challenges, including uncertainty control of model outputs, optimization of consistency among models, and effective integration of high-dimensional data. Future research could explore advanced techniques such as deep ensemble learning, Bayesian optimization, and model uncertainty assessment to further enhance the stability and generalization capability of decision-level fusion methods, and to advance the development of more robust and intelligent orchard yield estimation systems.

## 6 Orchard yield estimation platforms

With the rapid advancement of smart agriculture, technologies such as machine vision, remote sensing, and multi-sensor fusion have been widely applied in orchard yield estimation, effectively improving the accuracy of fruit recognition and counting while reducing errors caused by environmental factors. A normalized cross-study comparison of related research is presented in **Table 5**. These technologies rely on highly automated data acquisition platforms—such as intelligent systems integrating optical imaging, LiDAR, thermal infrared, and hyperspectral sensors—which significantly reduce manual involvement and enable all-weather, high-precision orchard monitoring.

**Table 5:** Normalized comparison of representative studies on fruit detection and yield estimation.

Fruit type	Data scale	Platform and Data type	Processing latency	Main advantage	Accuracy metric	Performance	Reference
Apple/Apple Blossom	3,200 images	Handheld RGB	14.2 ms/image	Lightweight U-Net variant, efficient flower detection	R²	0.80	Bhattarai et al. (2024)
Citrus	6 video sequences	UGV RGB	12 ms/frame	Combines LiDAR-based tree segmentation with CNN-based fruit recognition	AP, MAE	Detection: AP = 0.957; Counting: MAE = 0.081	Zhang et al. (2022a)
Mango	45 trees	UGV Hyperspectral + LiDAR	—	Combines LiDAR-based automatic tree segmentation with spectral CNN for fruit identification	R²	0.79	Gutiérrez et al. (2019)
Pitaya	2,000 plants	UAV RGB	—	Yield estimation under different growth scenarios	Acc	93.28%	Li et al. (2022b)
Ginkgo	Approx. 1,408 trees	UAV Hyperspectral	—	Enhanced UAV multispectral resolution improves yield prediction accuracy	R²	0.64	Fan et al. (2024)
Pear	Approx. 700 trees	Ground Hyperspectral	—	Analyzed temporal dependency between spectral vegetation indices and fruit yield/quality	R²	0.6–0.8	Van Beek et al. (2015)
Blueberry	2,500 images	UGV RGB	120 ms/image	High segmentation accuracy, capable of estimating ripeness	mAP	91.2%	Ni et al. (2020)
Apple	1,500 images/60 trees	UAV	30 ms/image	Combines Mask R-CNN with point cloud volume estimation	RMSE	6.42	Gené-Mola et al. (2020)
Citrus	3,600 images	UAV RGB	40 ms/image	Real-time detection under complex lighting conditions	mAP	91.23%	Mirhaji et al. (2021)
Litchi	1,800 images	UAV RGB	0.3 ms/image	Accurate inflorescence counting via multi-scale feature fusion	F1-score	0.89	Lin et al. (2024)
Passion Fruit	1,200 video segments	UGV	90 ms/frame	GhostNet-based high frame rate video detection	Acc	96%	Sun et al. (2024)
Multi-fruit dataset	4,000 images	Handheld RGB	0.25 ms/image	Semi-supervised approach with strong adaptive thresholding	mAP	88.7%	Mai et al. (2024)

Currently, orchard yield estimation platforms are mainly divided into three categories: handheld devices, unmanned ground vehicles (UGVs), and unmanned aerial vehicles (UAVs). Each platform offers unique advantages in terms of data acquisition methods, load capacity, and suitable application scenarios. Through coordinated deployment and integration of multiple platforms, an efficient and precise yield estimation system can be established, facilitating intelligent orchard management and the development of precision agriculture.

### 6.1 Handheld platforms

Handheld platforms are widely used in orchard yield estimation due to their relatively low cost and operational flexibility. During data acquisition, technicians manually operate sensor devices to capture images or videos of the tree canopy, and then process and analyze the collected data to achieve fruit counting and yield estimation for individual trees or entire orchards. In recent years, with the integration of mobile and cloud computing technologies, fruit counting methods based on smartphones and cloud platforms have become a research hotspot. For example, Fu et al. (2018) developed a kiwi fruit counting application based on the Android mobile operating system; field tests demonstrated that handheld platforms can achieve efficient yield estimation. Similarly, Woo et al. (2023) deployed the YOLOv5 model on smartphones, enabling berry yield estimation without the need for network connectivity and achieving a processing speed of 3 frames per second. Users can complete fruit counting simply by taking photos or videos around the tree. Roy et al. proposed a method for apple counting by capturing images with smartphones and employing deep learning models such as Mask R-CNN. Users take multi-angle images of fruit trees in the field, and the system automatically segments and counts the apples, enabling yield estimation at the single-tree and small-area levels. Experiments showed that this approach delivers good accuracy and practical applicability even in complex environments (Deep learning-based apple counting using Mask R-CNN for yield estimation).

The handheld device approach offers high flexibility in data acquisition, allowing users to adjust shooting angles and directions as needed to suit the fruit distribution characteristics of different crops. However, handheld devices remain limited in terms of automation and scalability for large-scale applications. Consequently, researchers have begun to explore automated data acquisition platforms such as ground vehicles and UAVs to improve the efficiency and accuracy of fruit counting and advance the development of intelligent orchard management.

### 6.2 Ground vehicle-based estimation platforms

**Figure 5:**
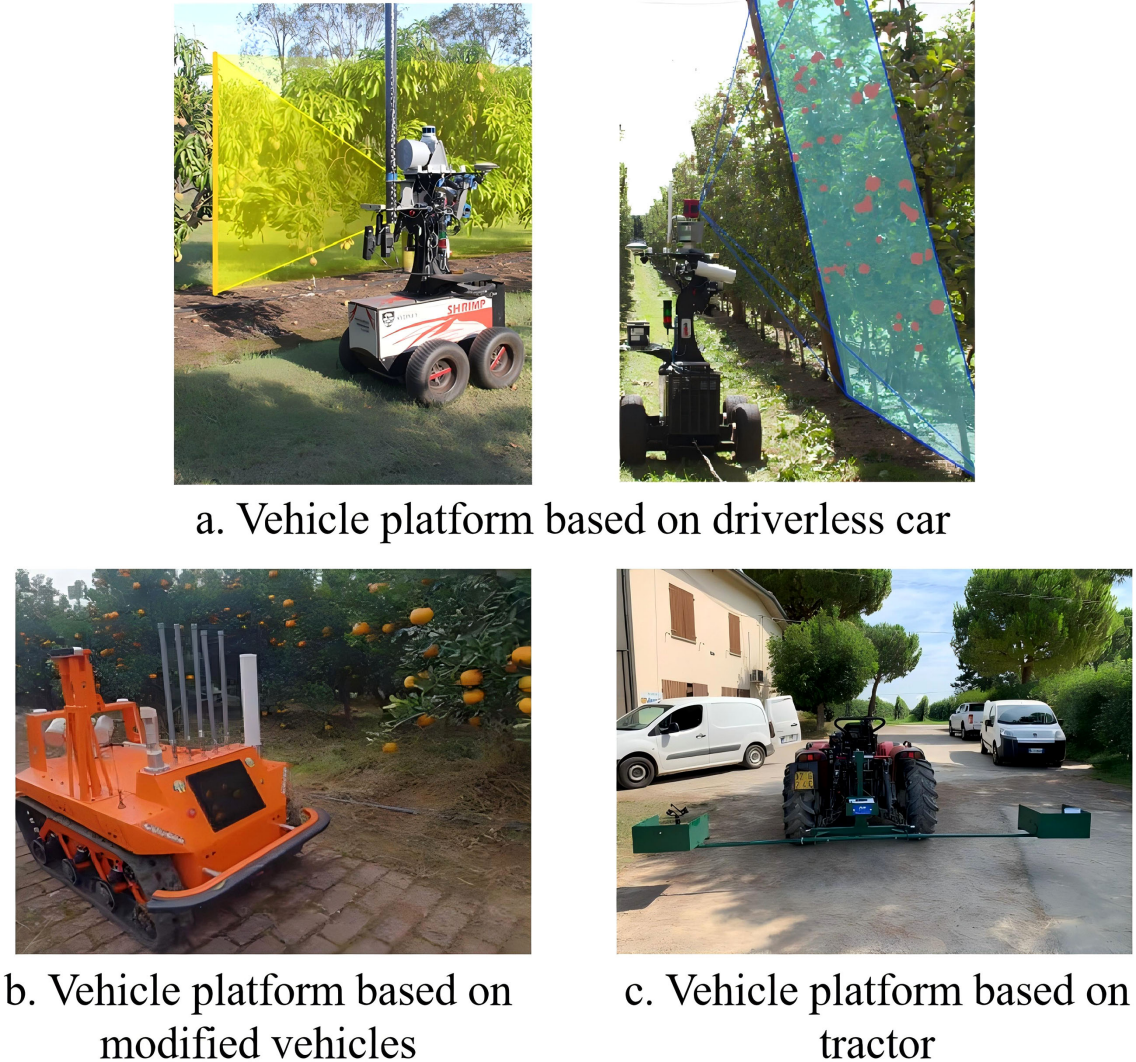
Ground vehicle sensor mounting platform. Examples of ground-based platforms used for orchard data acquisition: **(a)** vehicle platform based on a driverless car; **(b)** vehicle platform based on modified vehicles; **(c)** vehicle platform based on a tractor. These platforms can carry RGB, depth, LiDAR, and/or multispectral sensors to collect imagery relevant to yield estimation.

Data acquisition using handheld devices is labor-intensive and inefficient, making it difficult to meet the needs of yield estimation in large orchards. To address this challenge, researchers have proposed integrating sensors, Global Navigation Satellite System (GNSS), and navigation systems into tracked or wheeled ground vehicles for orchard yield estimation. Representative ground vehicle platforms are shown in **Figure 5**. Ground vehicles not only reduce manual labor but can also carry additional equipment, such as high-definition cameras, multispectral cameras, and supplementary lighting devices, thereby improving image quality and data processing capability. To date, numerous studies have utilized ground vehicles for data collection in orchard yield estimation, yielding promising results.

To date, several studies have employed ground vehicles for data collection in orchard yield estimation and have achieved promising preliminary results. For example, in research combining high-definition cameras with unmanned vehicles for video-based fruit detection and counting, Gao et al. (2022) developed an automated method for apple video detection by mounting a camera on an unmanned vehicle, recording videos, and calculating the reference displacement between consecutive frames using motion trajectory analysis. This approach achieved an apple counting accuracy of up to 91.49%. Zhang et al. (2022a) used unmanned vehicles equipped with cameras to collect video data for citrus yield estimation. In their study, YOLOv3 was used as the backbone model for fruit monitoring, and a tracking algorithm was proposed to address issues of occlusion and duplicate counting. In research combining spectral cameras with unmanned vehicles for video-based detection and counting, Gutierrez et al. (2019) used ground unmanned vehicles equipped with multispectral cameras to acquire hyperspectral images of mango trees and achieved mango counting through pixel-level data segmentation. During image recognition, uneven illumination is one of the most significant factors affecting detection performance. Therefore, some researchers have leveraged the high payload capacity of ground vehicles to carry supplementary lighting devices and thus improve the quality of collected data. In studies on imaging optimization with illumination compensation devices and unmanned vehicles, Mekhalfi et al. (2020) used a tractor platform equipped with cameras, GPS, and LED lights, powered by the vehicle, and ultimately achieved kiwi fruit yield estimation.

Accurate yield estimation at the single-tree level is fundamental for orchard yield assessment. However, due to variations in tree height and canopy size, fixed imaging parameters may result in: (1) distances that are too close, making it impossible to capture the entire canopy and affecting fruit counting; or (2) distances that are too far, potentially including ground or neighboring trees’ fruit, thus compromising estimation accuracy. To address this, some researchers have integrated GNSS, LiDAR, and visual sensors on unmanned vehicle platforms, achieving multi-sensor data fusion for yield estimation and navigation in complex orchard environments. For example, Islam et al. used unmanned vehicles equipped with different RTK systems, long-range wide area network receivers, and AI software to identify the number of peach, plum, and apricot fruits. During monitoring, they combined RGB-D cameras and LiDAR to control the distance between the platform and the fruit trees, effectively avoiding missed detections due to improper imaging angles.

At present, ground unmanned vehicles have become important tools for automated orchard yield estimation. The integration of high-definition cameras, multispectral cameras, and supplementary lighting can improve fruit counting accuracy. For complex orchard scenarios, unmanned vehicle systems equipped with LiDAR can optimize imaging parameters and enhance the precision of single-tree fruit counting.

### 6.3 Yield estimation using UAV platforms

**Figure 6:**
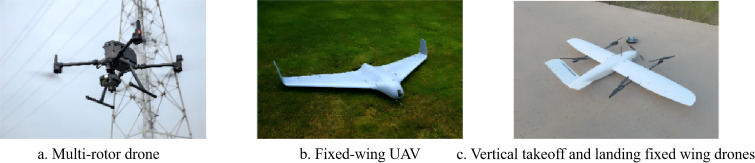
UAV sensor mounting platform. Examples of unmanned aerial vehicle (UAV) platforms used for orchard monitoring and yield estimation: **(a)** multi-rotor drone; **(b)** fixed-wing UAV; **(c)** vertical takeoff and landing fixed-wing drone.

Fruit trees are often cultivated in mountainous and hilly areas, where complex terrain imposes significant limitations on data acquisition by ground vehicles. In practical orchard yield estimation, UAVs (unmanned aerial vehicles) have emerged as highly advantageous remote sensing platforms due to their high mobility, wide imaging coverage, and efficient data collection capability, making them particularly suitable for rapid information acquisition in large-scale orchards. Currently, UAV platforms are mainly divided into multi-rotor, fixed-wing, and vertical takeoff and landing (VTOL) fixed-wing types. **Figure 6** shows three typical UAV platforms, and their technical characteristics and applicable scenarios are summarized in **Table 6**. By carrying multiple sensors and conducting low-altitude flights, UAVs can obtain high-resolution information on crop growth; subsequently, methods such as data preprocessing, image fusion, and machine learning algorithms are used to integrate multi-modal data into visualized yield prediction results.

**Table 6:** Technical characteristics and application scenarios of UAV platforms.

Category	Technical characteristics	Applicable scenarios
Multi-rotor unmanned aerial vehicle	It has a simple structure and flexible operation, capable of vertical take-off and landing as well as hovering, and is suitable for low-speed and small-scale operations.	It is suitable for fine operations in small orchards, such as plant protection spraying, pest and disease monitoring, and orchard yield estimation, especially for environments with complex terrain and limited space
Fixed-wing unmanned aerial vehicle	It has a fast flight speed, a long range and strong endurance, and is suitable for large-scale cruise missions.	It is suitable for monitoring tasks in large-scale farmlands, orchards or forests, such as long-distance patrols, environmental assessment, meteorological monitoring, etc., and is suitable for open terrain.
Multi-rotor and vertical take-off and landing fixed-wing unmanned aerial vehicles	Combining the advantages of multi-rotor and fixed-wing aircraft, it can not only take off and land vertically but also conduct efficient cruising, making it suitable for long-distance missions	It is suitable for application scenarios that require long battery life and complex environments, such as large-scale orchard monitoring, agricultural mapping, logistics transportation, etc

On UAV platforms, conventional visible-light cameras are the most widely used sensors. For example, Li et al. (2023) mounted sensors on UAVs to capture images of longan tree canopies and proposed a two-stage yield estimation method: in the first stage, a target detection model is used to identify the number of longan fruits in images; in the second stage, a regression model is constructed to fit the number of detected fruits with the actual count, enabling longan yield estimation using only canopy top-view images. Similarly, Subeesh et al. (2024) used UAVs equipped with YOLO object detection networks to identify citrus fruit on trees, and further integrated the detection model into a cloud server for greater user convenience. Their research also distinguished between mature and immature fruit and used deep learning models to segment and estimate fruit size. However, the yield estimation methods in these studies typically rely on counting fruit per tree and extrapolating by the total number of trees, which is relatively inefficient. To improve estimation efficiency, some researchers have adopted sampling estimation methods, whereby yield is measured for a subset of sampled trees and then extrapolated to the entire orchard. Uribeetxebarria et al. (2019) collected canopy images of peach orchards using UAVs and applied ranked set sampling to estimate yield, achieving orchard yield estimation using only 50% of the sample size.

In addition to vision-based yield estimation, UAV remote sensing platforms can carry a variety of advanced sensors to capture more comprehensive crop growth information. Currently, the main types of remote sensing sensors include multispectral and hyperspectral cameras. In recent years, some researchers have also explored thermal imaging for yield estimation. Thermal imaging can capture real-time temperature distribution on crop surfaces, indirectly reflecting plant water stress and transpiration status, and offering new perspectives for crop growth assessment and yield prediction. For example, Caruso et al. (2023) used UAV-mounted thermal imagers to detect internal temperature distribution of olive trees and generate the Crop Water Stress Index (CWSI), with studies showing a negative correlation between CWSI and olive yield. This research demonstrates the potential of thermal imaging for fruit tree yield estimation. Although thermal infrared sensors are currently less commonly applied in fruit tree yield estimation, some researchers have explored their use for crop yield estimation in soybean, wheat, and other crops, laying a technical foundation for subsequent extension to forestry applications.

With ongoing advances in UAV technology, an increasing variety of sensors—including LiDAR, thermal infrared sensors, and multi-angle imaging systems—are being integrated into UAV platforms. These sensors not only facilitate fruit yield estimation but also capture additional information such as tree height, canopy structure, and phenotypic traits, thereby providing more comprehensive and multi-dimensional data support for fruit yield prediction.

## 7 Existing challenges

With the rapid advancement of smart agriculture, orchard management is increasingly moving towards digitalization and intelligence. Various modern monitoring technologies have been widely adopted for orchard yield estimation. However, due to complex orchard terrain, remote locations, and inconsistent management practices, current yield estimation technologies face certain limitations, and a highly automated and standardized application system has yet to be developed. The specific issues include:

### 7.1 Challenges in yield estimation during flowering and young fruit stages

Yield estimation during the flowering and young fruit stages is highly uncertain because the fruits are immature and susceptible to various environmental factors. Specifically, key issues include:

Firstly, uncertainties related to natural pollination, extreme weather conditions (e.g., low temperatures, heavy rainfall, wind damage), and pests and diseases frequently result in substantial fruit drop. Consequently, the actual yield is often significantly lower than the potential yield estimated during early stages, leading to widespread overestimation and reducing the accuracy and reliability of predictions. Secondly, during flowering and young fruit stages, fruits are small, pale-colored, and visually similar to leaves and branches, presenting challenges for current machine vision technologies, resulting in frequent misdetections and missed detections, further lowering yield estimation accuracy.

### 7.2 Limitations and poor synergy of existing remote sensing sensors

Currently, multispectral remote sensing is predominantly used for orchard yield estimation. However, multispectral imagery is limited by the number of spectral bands and spatial resolution, making it difficult to comprehensively and precisely capture subtle physiological changes in fruit growth. These limitations significantly affect the accuracy and applicability of yield estimation models.

Moreover, although LiDAR and thermal infrared imaging sensors, providing detailed information on crop structure, temperature, and three-dimensional morphology, have been widely employed in field crop yield estimation, studies integrating these sensors for orchard yield estimation remain scarce.

### 7.3 Insufficient fruit detection accuracy and challenges under occlusion conditions

The lack of robustness in fruit detection models significantly hampers precision improvement. Yield estimation in orchards can be categorized into early-stage and late-stage estimates. Early-stage estimates aim to optimize thinning and precise fertilization and irrigation, while late-stage estimates assist economic assessments and market planning before harvest. Due to the considerable variation in fruit morphology, color, and distribution across growth stages, models require enhanced robustness to improve detection accuracy. Despite advancements in deep learning-based image recognition, model generalization remains inadequate under complex orchard conditions.

In orchard environments, fruits frequently experience complex occlusions by leaves, branches, and other fruits, causing incomplete target fruit information in captured images and significantly challenging precise fruit detection. Furthermore, fruit tree diversity and structural differences result in varying degrees of occlusion, making it difficult for conventional imaging methods to comprehensively capture effective fruit information within occluded areas. Consequently, most existing yield estimation models rely on idealized or standardized orchard conditions, severely limiting model generalization and practical application effectiveness due to complex occlusion scenarios.

### 7.4 Limited application of multi-source data fusion techniques

Multi-source data fusion is crucial for improving orchard yield estimation accuracy, yet its application remains in the early stages of research. Current multi-source data fusion methods mainly involve simple concatenation or superimposition of orchard characteristics, lacking deep exploration and analysis of inherent data associations. Consequently, the potential advantages of multi-source data fusion have not been fully realized, constraining improvements in yield estimation accuracy.

Moreover, the significant heterogeneity in multi-source data complicates effective integration. Specifically, remote sensing imagery (e.g., satellite, UAV), ground sensor data (e.g., visual and environmental sensors), and physiological health monitoring data differ markedly in spatial scales, temporal resolutions, data formats, and modalities. These discrepancies intensify the complexity of data fusion and comprehensive analysis. Additionally, data quality varies considerably among sources, with prevalent noise and uncertainty complicating traditional fusion methods, further reducing the reliability and stability of integrated data.

### 7.5 Limited automation and scene adaptability

Currently, research on orchard yield estimation primarily focuses on theoretical analyses based on existing multi-source data (e.g., imagery, point clouds, meteorological data) processing, aimed at data fusion and model construction. Although initial progress has been made in data processing and algorithm development, these processes still rely heavily on manual intervention, resulting in inadequate automation and intelligence levels overall.

Furthermore, current data collection platforms typically target specific orchard scenarios, whereas most orchards in China are situated in complex mountainous or hilly terrains with diverse planting structures, variable terrain conditions, and uneven planting densities. Consequently, technological systems designed for single orchard types encounter significant obstacles during practical implementation. Besides terrain challenges, difficulties in navigation, constrained path planning, and unreliable signal transmission in complex orchard environments severely limit the stability and reliability of unmanned equipment, increasing implementation difficulty and associated risks.

## 8 Future prospects

**Figure 7:**
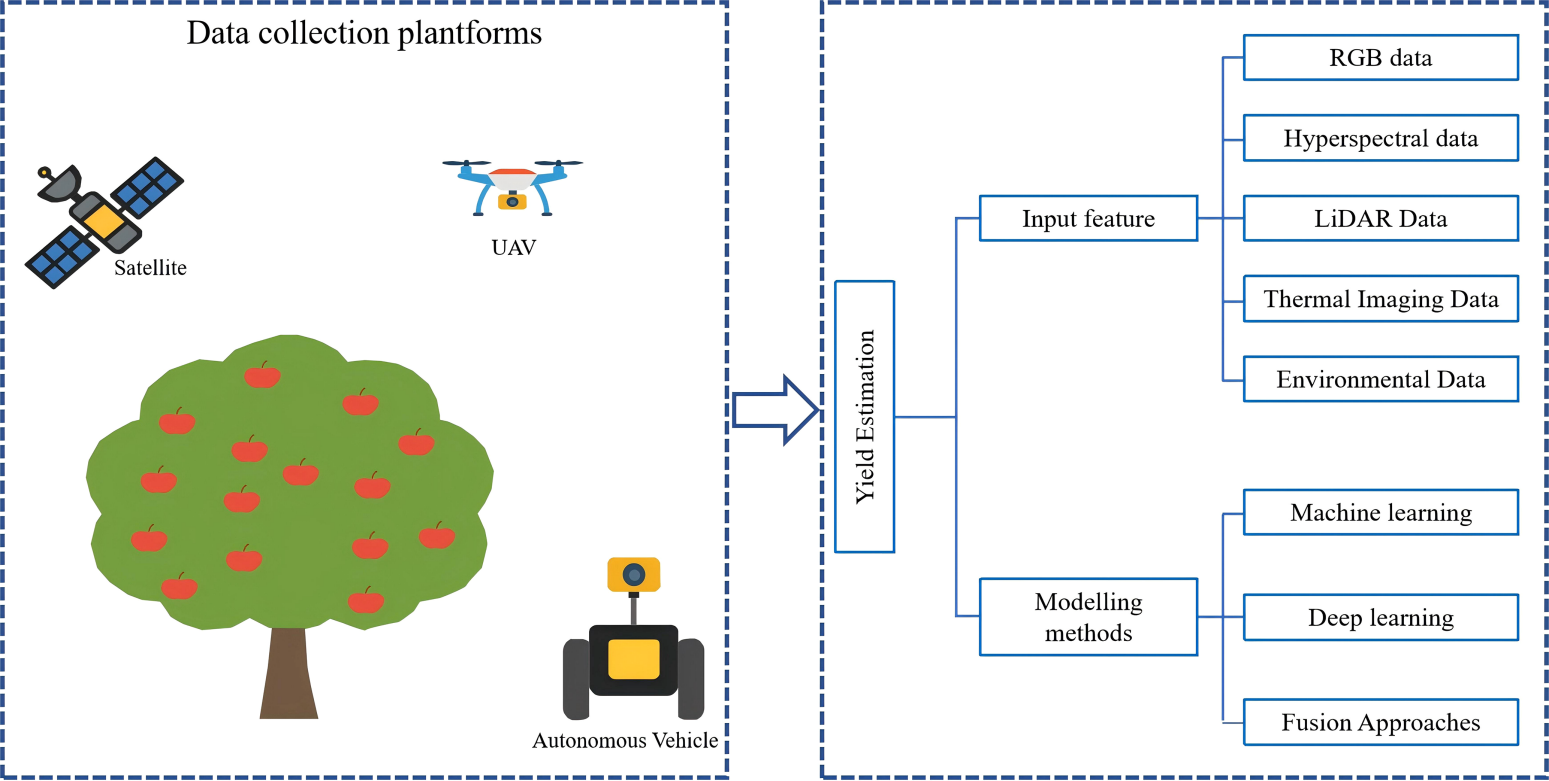
Framework of a modern fruit tree yield estimation system. This end-to-end workflow includes multi-platform data acquisition, data preprocessing, feature extraction, yield estimation (identification-based counting and/or regression-based modeling), and output products (e.g., per-tree yield estimates and orchard-level yield distribution maps) to support management decision-making.

With the rapid development of smart agriculture and forestry, orchard management is gradually advancing toward digitalization and intelligence. Various modern monitoring technologies have been successively applied to orchard yield estimation research, with both application directions and methodologies continuously expanding, leading to significant progress in yield estimation. From the perspective of estimation technology, machine vision and remote sensing have been widely adopted for yield estimation in different types of orchards. In terms of estimation approaches, both direct and indirect yield estimation methods have been developed to meet the needs of various application scenarios for different fruit tree species. In future research, it will be essential to fully leverage advanced technologies such as artificial intelligence, remote sensing, the Internet of Things (IoT), and big data to develop integrated ground–air, multi-platform yield estimation systems. These systems should facilitate the fusion of multi-source data to achieve automated and modernized orchard yield estimation. **Figure 7** illustrates the developmental objectives and technical framework of future orchard yield estimation systems. On this basis, this paper discusses the challenges and potential development directions of modern orchard yield estimation from the following four perspectives:

### 8.1 The need for establishing large-sample, long-term observation databases

One of the prominent challenges currently facing orchard yield estimation research is the lack of comprehensive, large-sample, and long-term observation databases. Accurate early-stage yield prediction largely depends on extensive and multidimensional datasets that record the dynamic growth processes of fruit trees under different temporal and environmental conditions. However, most existing studies are limited by small sample sizes, short observation periods, or data collected from a single orchard or variety, which greatly restricts the representativeness and generalizability of prediction models.

Establishing a large-scale database that continuously collects and archives data on fruit tree growth, flowering, fruit set, young fruit development, and final yield across different periods, orchards, varieties, and management practices would provide a more robust foundation for early-stage yield prediction. Such a database would enable the detailed analysis of the temporal and spatial dynamics of fruit tree development, facilitating the identification of key factors influencing yield formation. For example, systematically documenting the relationship between flowering density and final yield would allow for more accurate and timely yield estimation at early growth stages, ultimately supporting better orchard management and decision-making.

### 8.2 Zero-shot models and the challenge of occluded fruit recognition in orchards

To address the issue of insufficient model robustness, future research can focus on improving model architectures and training methodologies. For instance, integrating attention mechanisms with graph convolutional networks may guide the model to infer the potential locations of occluded fruits based on spatial relationships, effectively reducing missed and false detections. In addition, employing emerging techniques such as generative adversarial networks (GANs), domain adaptation, and few-shot learning can help mitigate the adverse effects of insufficient data on overall model accuracy. It is also promising to design models with transfer learning or lifelong learning capabilities, enabling continuous adaptation to changes in fruit morphology and thereby enhancing recognition accuracy across different growth stages and cultivars.

In recent years, detection models based on visual Transformers, such as DINO, and further advancements like Grounding DINO, have demonstrated outstanding performance in complex object detection tasks. Grounding DINO achieves robust zero-shot learning by deeply integrating textual semantics and visual features, enabling the detection of various fruit species and categories even in the absence of specifically annotated samples. This capability significantly reduces dependence on large-scale labeled datasets and lowers training costs, which is particularly valuable for orchards characterized by diverse cultivars and limited data availability. Future studies on fruit yield estimation can further explore and optimize Grounding DINO-based zero-shot learning frameworks to realize more generalizable and intelligent fruit detection systems. Therefore, developing zero-shot fruit detection based on Grounding DINO, thereby reducing data annotation and training costs, represents a key research direction for the future of orchard yield estimation.

To address the challenges of fruit occlusion by leaves and branches, intelligent orchard perception platforms based on 3D reconstruction and digital twin technologies can be developed. By integrating image data from multiple perspectives—such as unmanned aerial vehicles, mobile robots, and fixed cameras—these platforms can alleviate the blind spots caused by structural occlusions inherent in traditional single-view imaging. Additionally, the integration of airflow-assisted devices (e.g., small fans) into data acquisition platforms can dynamically reduce leaf occlusion during image collection, thereby improving fruit visibility and recognition accuracy.

### 8.3 Constructing a multi-source data fusion framework

Current research has demonstrated that multimodal data fusion can significantly improve the accuracy and stability of agricultural yield prediction. For example, by combining satellite remote sensing imagery and climate-meteorological data, it is possible to analyze crop growth dynamics, environmental condition changes, and crop stress more precisely, thereby enabling more refined crop yield prediction. The fusion of LiDAR point cloud data and hyperspectral remote sensing data not only enhances the recognition of crop canopy structure and physiological characteristics but also reduces the limitations of traditional single-source prediction methods, thereby improving the robustness and generalizability of prediction results. In the application of multi-source data fusion for orchard yield estimation, the latest research progress in agricultural science should be fully referenced, especially achievements in crop yield prediction model construction, remote sensing technology application, and climate and soil factor analysis for crops such as wheat and soybean (Maimaitijiang et al., 2020; Li et al., 2022a; Zhang et al., 2024).

In summary, future research on orchard yield estimation should aim to build a rich and efficient multi-source heterogeneous data fusion system, expanding and optimizing data acquisition channels. This includes the effective integration of satellite remote sensing imagery, UAV aerial photography, LiDAR point clouds, hyperspectral imaging, and ground-based sensors (such as temperature, humidity, and soil sensors) to fully exploit the potential of collaborative sensing and synergistic effects of multi-source data. Furthermore, relevant auxiliary information affecting fruit growth, such as pest and disease monitoring data, meteorological data, and crop physiological indicator data, should be incorporated to comprehensively enhance the perception dimensions and environmental adaptability of data-driven yield estimation models.

### 8.4 Promoting the construction of unmanned smart orchards

The development of unmanned orchard yield estimation systems requires deep integration from theory to practice. On one hand, efforts should focus on the research and development of intelligent data acquisition platforms and sensor systems with strong cross-scenario adaptability. For example, modular unmanned systems equipped with multimodal sensors, combined with high-precision autonomous navigation, real-time obstacle avoidance, and intelligent adaptive path planning, can realize automated data acquisition in complex scenarios. On the other hand, a real-time data analysis and processing platform integrating edge computing and cloud computing should be constructed to promote the integration and intelligence of data acquisition, transmission, processing, and decision-making processes. At the same time, unified standards for data, equipment, and evaluation should be actively promoted, and a standardized system for orchard yield estimation applicable to diverse scenarios should be gradually established, providing effective theoretical and technical support for the implementation of unmanned orchard yield estimation.

Specifically, future research should further strengthen the development of autonomous navigation systems for unmanned platforms centered on Simultaneous Localization and Mapping (SLAM) technology. By leveraging LiDAR, visual perception, inertial navigation, and multi-sensor fusion technologies, the environmental perception and autonomous navigation capabilities of unmanned platforms can be enhanced, reducing the impact of complex orchard environments (such as irregular planting areas, challenging terrain, and occlusions) on operational accuracy and stability, and promoting unmanned platforms towards high-precision autonomous operations. Meanwhile, with the large-scale data storage and rapid processing capabilities of cloud computing platforms, and the efficient analysis and deep mining of real-time monitoring data enabled by artificial intelligence (AI), high-precision and timely orchard yield distribution maps can be generated.

### 8.5 Cross-season information fusion strategy from dormant to canopy stages

The dormant stage marks the beginning of the annual growth cycle of fruit trees and serves as a critical period for cross-season yield prediction. During this phase, the trees are leafless, and branches and flower buds are fully exposed, allowing for accurate structural measurement and assessment of potential fruiting capacity. By utilizing high-resolution RGB or LiDAR imaging, it is possible to acquire three-dimensional (3D) canopy point clouds for the automatic identification of branch volume, branching density, and flower bud counts, thereby providing valuable prior information for yield prediction during the flowering and fruiting stages. Compared with in-season monitoring, imagery collected during dormancy is less affected by illumination variability and occlusion, resulting in higher data consistency and improved potential for developing stable multi-year yield estimation models. Recent studies, for example, have estimated branch-level yield based on branch orientation and thickness during dormancy (Ahmed et al., 2025).

With the advancement of sensing and computational technologies, research has increasingly focused on integrating monitoring data from the dormant stage and the early green fruit stage to enable continuous assessment from “potential yield” to “actual fruit load.” The early green fruit stage represents the period between post-flowering and the onset of rapid fruit expansion, during which fruits are small but detectable. By comparing flower bud counts obtained during the dormant stage with fruit recognition results from the early green fruit stage, key indicators such as fruit set rate and early fruit drop rate can be quantified. This cross-season information fusion significantly enhances the accuracy of early-season yield prediction, bridging the gap between pre-flowering potential and realized productivity.

## 9 Conclusion

This study provides a systematic review of recent advances and emerging trends in orchard yield estimation based on multi-source sensing technologies. With the rapid development of machine vision, spectral remote sensing, and multi-sensor fusion, fruit yield estimation has gradually transitioned from manual experience-based assessment to data-driven and model-based prediction. Significant progress has been achieved in fruit recognition, counting, and size quantification, substantially enhancing the automation and intelligence of orchard yield estimation. These advances have established a solid foundation for the digitalization and precision management of the fruit industry.

Despite these achievements, current approaches still face several challenges, including severe occlusion, environmental variability, limited cross-season generalization, and high data heterogeneity. The integration of multimodal sensing technologies—such as RGB imaging, LiDAR, hyperspectral, and thermal infrared sensing—offers new opportunities to overcome these limitations. In particular, monitoring during both the dormant and early green fruit stages provides crucial support for cross-season yield continuity, enabling dynamic yield modeling across the entire phenological cycle from bud formation to harvest.

Future research on orchard yield estimation should focus on establishing a unified intelligent sensing framework that enables deep fusion of multi-source data through coordinated observations from ground, aerial, and satellite platforms. By integrating artificial intelligence-driven predictive analytics, yield estimation systems can achieve greater scalability and adaptability. Furthermore, zero-shot and few-shot detection methods based on vision–language models (e.g., Grounding DINO) will significantly reduce annotation costs while enhancing model transferability and cross-variety adaptability. The incorporation of real-time data acquisition via unmanned ground vehicles (UGVs) and unmanned aerial vehicles (UAVs), combined with edge computing and cloud-based processing, will further accelerate the transformation of orchard yield monitoring toward a fully automated, unmanned, and intelligent paradigm.

## Data Availability

The raw data supporting the conclusions of this article will be made available by the authors, without undue reservation.

## Glossary

AI: Artificial Intelligence
BP: Back Propagation (Neural Network)
CCVI: Color Change Vegetation Index
CWSI: Crop Water Stress Index
DL: Deep Learning
ECA: Efficient Channel Attention
FPS: Frames Per Second
GAN: Generative Adversarial Network
GNDVI: Green Normalized Difference Vegetation Index
GNSS: Global Navigation Satellite System
IoU: Intersection over Union
KNN: K-Nearest Neighbors
LAI: Leaf Area Index
LED: Light Emitting Diode
LSTM: Long Short-Term Memory
mAP: mean Average Precision
NDVI: Normalized Difference Vegetation Index
NDVIre: Normalized Difference Vegetation Index of Red Edge
PANet: Path Aggregation Network
PCA: Principal Component Analysis
PLS: Partial Least Squares
RGB: Red Green Blue
RGB-D: Red Green Blue and Depth
RR: Ridge Regression
RTK: Real-Time Kinematic
R-CNN: Region-based Convolutional Neural Network
SORT: Simple Online and Real-time Tracking
SVM: Support Vector Machine
UAV: Unmanned Aerial Vehicle
UGV: Unmanned Ground Vehicle
VTOL: Vertical Takeoff and Landing
YOLO: You Only Look Once.
